# MRE11 promotes oral cancer progression through RUNX2/CXCR4/AKT/FOXA2 signaling in a nuclease-independent manner

**DOI:** 10.1038/s41388-021-01698-5

**Published:** 2021-04-29

**Authors:** Yen-Yun Wang, Yuk-Kwan Chen, Steven Lo, Tsung-Chen Chi, Yi-Hua Chen, Stephen Chu-Sung Hu, Ya-Wen Chen, Shih Sheng Jiang, Fang-Yu Tsai, Wangta Liu, Ruei-Nian Li, Ya-Ching Hsieh, Chih-Jen Huang, Shyng-Shiou F. Yuan

**Affiliations:** 1 School of Dentistry, College of Dental Medicine, Kaohsiung Medical University, Kaohsiung, Taiwan; 2 Translational Research Center, Kaohsiung Medical University Hospital, Kaohsiung, Taiwan; 3 Department of Medical Research, Kaohsiung Medical University Hospital, Kaohsiung, Taiwan; 4 Center for Cancer Research, Kaohsiung Medical University, Kaohsiung, Taiwan; 5 Division of Oral Pathology & Maxillofacial Radiology, Kaohsiung Medical University Hospital, Kaohsiung, Taiwan; 6 Oral & Maxillofacial Imaging Center, College of Dental Medicine, Kaohsiung Medical University, Kaohsiung, Taiwan; 7 College of Medical, Veterinary and Life Sciences, University of Glasgow, Glasgow, UK; 8 Department of Dermatology, College of Medicine, Kaohsiung Medical University, Kaohsiung, Taiwan; 9 Department of Dermatology, Kaohsiung Medical University Hospital, Kaohsiung, Taiwan; 10 National Institute of Cancer Research, National Health Research Institutes, Miaoli, Taiwan; 11 Department of Biotechnology, College of Life Science, Kaohsiung Medical University, Kaohsiung, Taiwan; 12 Department of Biomedical Science and Environmental Biology, Kaohsiung Medical University, Kaohsiung, Taiwan; 13 Institute of Cancer Sciences, University of Glasgow, Glasgow, UK; 14 Department of Radiation Oncology, Kaohsiung Medical University Hospital, Kaohsiung, Taiwan; 15 Department of Radiation Oncology, Faculty of Medicine, College of Medicine, Kaohsiung Medical University, Kaohsiung, Taiwan; 16 Department of Obstetrics and Gynecology, Kaohsiung Medical University Hospital, Kaohsiung, Taiwan; 17 Graduate Institute of Medicine, College of Medicine, Kaohsiung Medical University, Kaohsiung, Taiwan; 18 Department of Biological Science and Technology, College of Biological Science and Technology, National ChiaoTung University, Hsinchu, Taiwan; 19 Center For Intelligent Drug Systems and Smart Bio-devices (IDS2B), National Chiao Tung University, Hsinchu, Taiwan

**Keywords:** Oral cancer, Double-strand DNA breaks

## Abstract

MRE11, the nuclease component of RAD50/MRE11/NBS1 DNA repair complex which is essential for repair of DNA double-strand-breaks in normal cells, has recently garnered attention as a critical factor in solid tumor development. Herein we report the crucial role of MRE11 in oral cancer progression in a nuclease-independent manner and delineate its key downstream effectors including CXCR4. MRE11 expression in oral cancer samples was positively associated with tumor size, cancer stage and lymph node metastasis, and was predictive of poorer patient survival and radiotherapy resistance. MRE11 promoted cell proliferation/migration/invasion in a nuclease-independent manner but enhanced radioresistance via a nuclease-dependent pathway. The nuclease independent promotion of EMT and metastasis was mediated by RUNX2, CXCR4, AKT, and FOXA2, while CXCR4 neutralizing antibody mitigated these effects in vitro and in vivo. Collectively, MRE11 may serve as a crucial prognostic factor and therapeutic target in oral cancer, displaying dual nuclease dependent and independent roles that permit separate targeting of tumor vulnerabilities in oral cancer treatment.

## Introduction

Oral squamous cell carcinoma (OSCC) is the 6th most common cancer worldwide [1], and it is particularly prevalent in Southeast Asian countries, with Taiwan reporting the highest global incidence [2, 3]. Despite diagnostic and therapeutic advances, 5-year survival globally remains at ~50% [1].

Recently MRE11, the nuclease component of the RAD50/MRE11/NBS1 (MRN) nuclease complex, has attracted attention as a potential key factor in the growth, invasion, and metastasis of a number of solid tumors including breast, lung, ovarian, and colorectal cancer. MRE11 is intimately involved in the DNA damage response, preserving genomic integrity via both Homologous Recombination (HR) and nonhomologous end joining pathways [4–9]. Although MRE11 is essential for protection of genomic stability, with nuclease activity of MRE11 shown to facilitate protection against oncogene induced replication stress in B lymphocytes [10], it may likewise exhibit maladaptive effects in the protection of established tumors from exogenous and endogenous sources of DNA damage. The clastogenic effect of ionizing radiation (IR) and chemotherapy is impaired in high MRE11 expressing phenotypes of breast and lung cancers, with recent data suggesting that high MRE11 expression lung cancer phenotypes may be protected from endogenous tumor related replication stress [11]. Furthermore, our previous study has shown that high MRE11 expression in breast cancer tissues was associated with more malignant behavior in breast cancer [11], whilst conversely MRE11 deficiency was associated with better disease-free and overall survival and improved treatment response to chemotherapy in colorectal cancer [12]. However, there are conflicting data regarding the role of MRE11 in carcinogenesis, with other studies finding that MRE11 may inhibit rather than promote oncogene driven tumorigenesis and metastasis [13]. This may reflect its complex and incompletely understood role in both protection of normal cell phenotypes from genomic instability, and the facilitation of cancer cell survival in the face of exogenous and endogenous DNA damage.

Nonetheless, the role of MRE11 in oral cancer remains to be elucidated. In this study, we explored the role of MRE11 in oral cancer behavior in vitro and in vivo, and examined whether MRE11 nuclease activity remains critical to oral cancer progression. This was prompted by the observation on cDNA microarray screening that MRE11 RNA expression was significantly elevated in oral cancer tissues compared to adjacent noncancerous oral tissues in the same patients (data not shown). Furthermore, we addressed the possibility that elevated MRE11 expression in oral cancer tissues may be mediated by replication stress in the tumor microenvironment.

## Results

### Elevated MRE11 expression promoted malignant oral cancer cell behavior

**Fig. 1:**
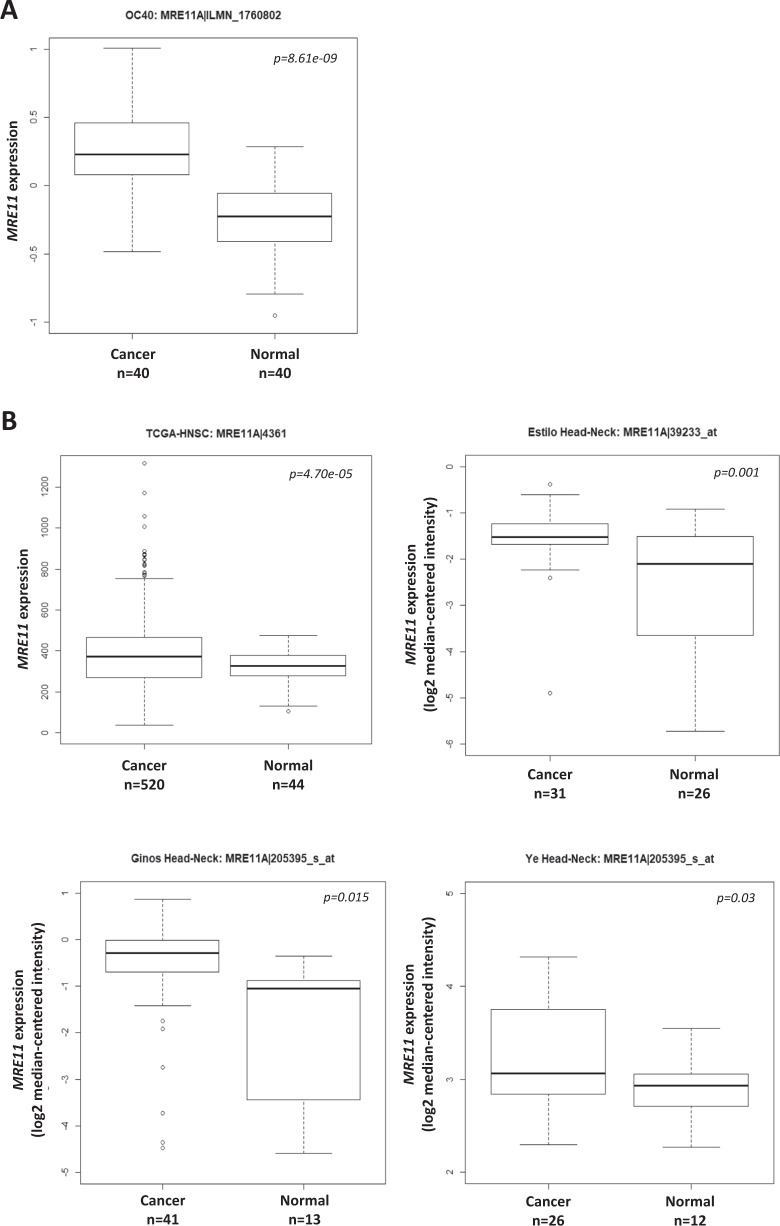

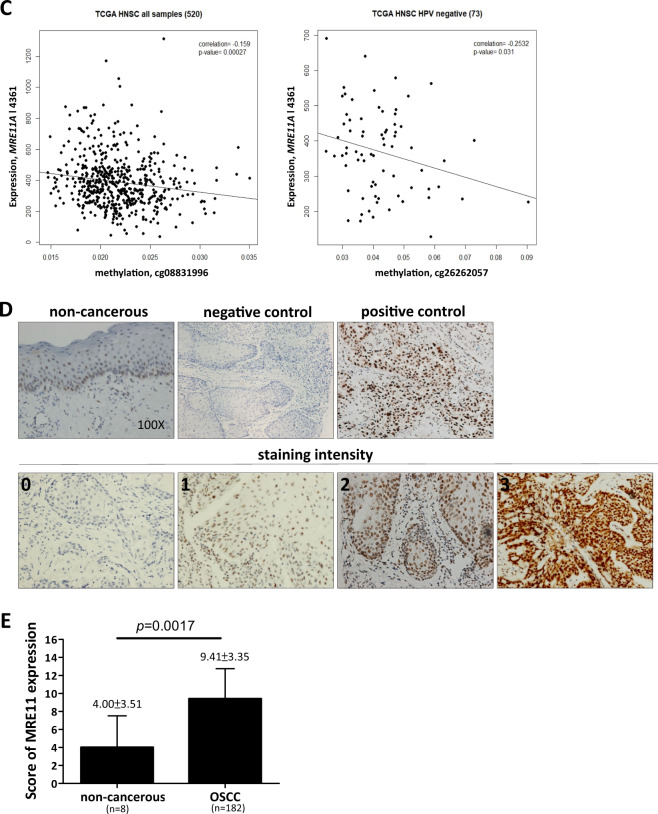

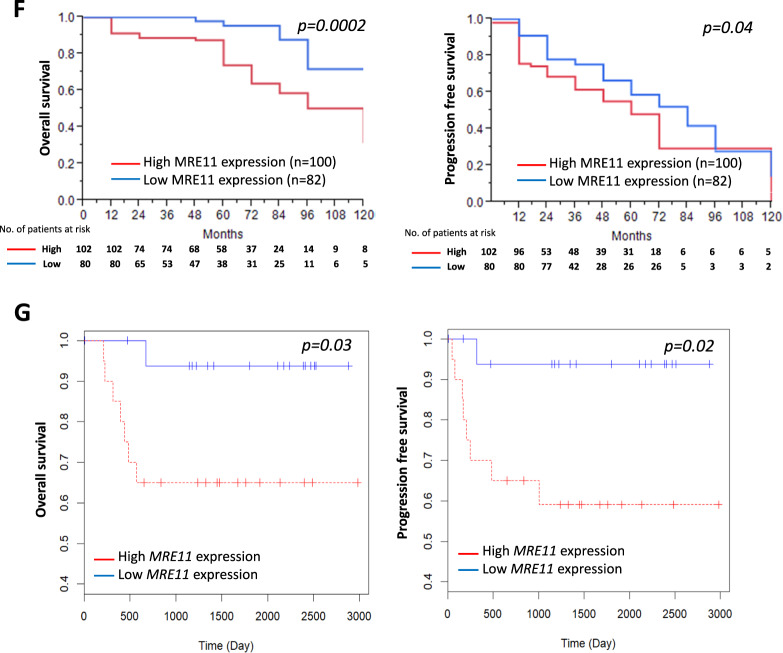
Elevated MRE11 expression in oral cancer tissues is associated with decreased overall and progression-free survivals. **A** Increased *MRE11* mRNA expression in oral cancer tissues, in comparison to normal tissues, from our database. **B** Increased *MRE11* mRNA expression in oral cancer tissues, in comparison to normal tissues, reported in online databases. **C** A negative correlation between *MRE11A* mRNA level and DNA methylation level in the putative promoter region of *MRE11*, reported in an online database. **D** Immunohistochemical staining for MRE11 protein expression in oral noncancerous and cancer tissues. **E** Quantitative result for MRE11 expression in oral noncancerous and cancer tissues. **F** Overall survival and progression-free survival for high and low MRE11 protein expression groups from our dataset. **G** Overall survival and progression-free survival for high and low *MRE11* mRNA expression groups reported in an online database.

To evaluate whether the expression of MRE11 is dysregulated in OSCC, we analyzed its expression in our OSCC gene expression dataset and found that *MRE11* mRNA is upregulated in oral cancer tissues (T), in comparison to oral noncancerous tissues (N) (Fig. 1A). Further analyses using publicly available online databases showed similar results (Fig. 1B). To assess whether *MRE11* mRNA expression is epigenetically regulated, we analyzed the correlation between the DNA methylation level of *MRE11* and its mRNA expression level in the TCGA-HNSC dataset. A significantly negative correlation was observed between the *MRE11* mRNA level and DNA methylation level of a CpG site (cg26262057) at the putative promoter region of *MRE11*, regardless of whether all samples (*n* = 520, *r* = −0.16, *p* < 0.001) or only HPV-negative samples (*n* = 73, *r* = −0.25, *p* = 0.03) were included (Fig. 1C), suggesting that *MRE11* transcription may be regulated by DNA methylation.

To confirm whether expression of MRE11 protein was also elevated in oral cancer tissues, immunohistochemical analysis was performed and the results showed that MRE11 expression is relatively low in normal oral epithelium compared to its expression level in oral cancer tissues (Fig. 1 D and E) (*p* = 0.0017). Further survival analyses according to MRE11 protein expression in oral cancer tissues showed that the high MRE11 expression group had decreased overall and progression-free survivals with *p* values of 0.0002 and 0.04, respectively (Fig. 1F). In agreement with these results, an online database also confirmed that high MRE11 mRNA expression was associated with decreased overall and progression-free survivals with *p* values of 0.03 and 0.02, respectively (Table S1 and Fig. 1G).

The above clinical observations prompted us to study whether MRE11 expression levels influenced oral cancer cell behaviors. We first determined the transwell migration activity in oral cancer cell lines. As shown in Fig. S1A, HSC-3 and OEC-M1 cells exhibited higher migration activity than CAL27 and CA9–22 cells. To downregulate the expression of MRE11 in oral cancer cells, we screened 4 different lentiviral clones and clone 1 showed the highest knockdown efficiency and was used in later knockdown studies (Fig. S1B). We also confirmed the knockdown efficiency of clone 1 by qRT-PCR (Fig. S1C). Using lentiviral knockdown and overexpression approaches, we decreased the expression of MRE11 in HSC-3 and OEC-M1 cells which have higher endogenous MRE11 expression, while we increased MRE11 expression in CAL 27 and CA9–22 cells which have lower endogenous MRE11 expression (Fig. S1D). After MRE11 knockdown, cell viability was decreased in oral cancer cells (Fig. S1E), accompanied by decreased expression of phospho-AKT(S473) and phospho-ERK1/2 (Fig. S1F). Knockdown of MRE11 also led to decreased colony formation in oral cancer cells (Fig. S1G). However, MRE11 overexpression did not lead to increased cell viability in oral cancer cells (Fig. S1E).

**Fig. 2:**
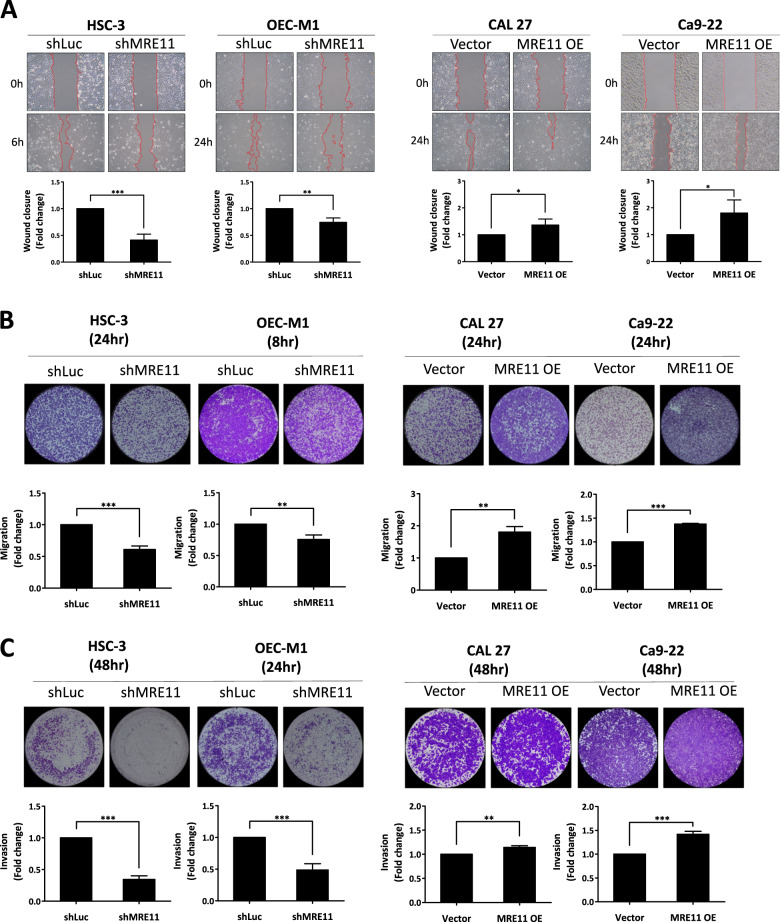

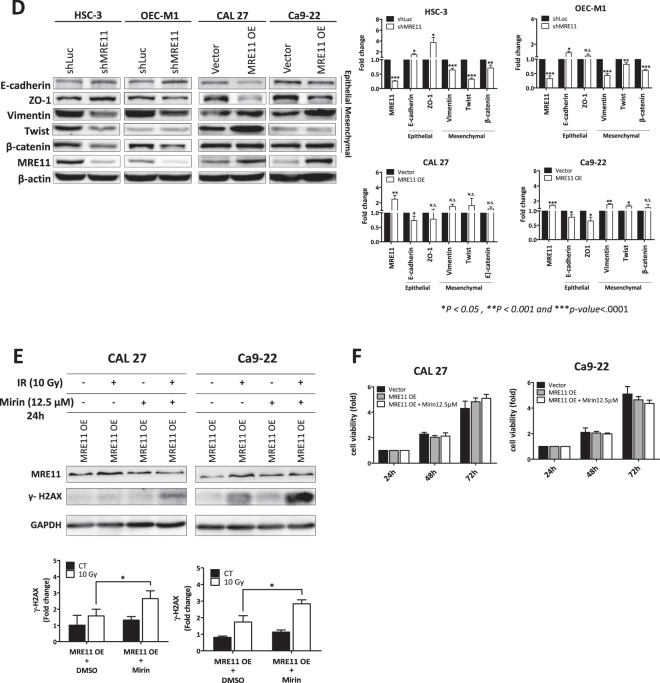

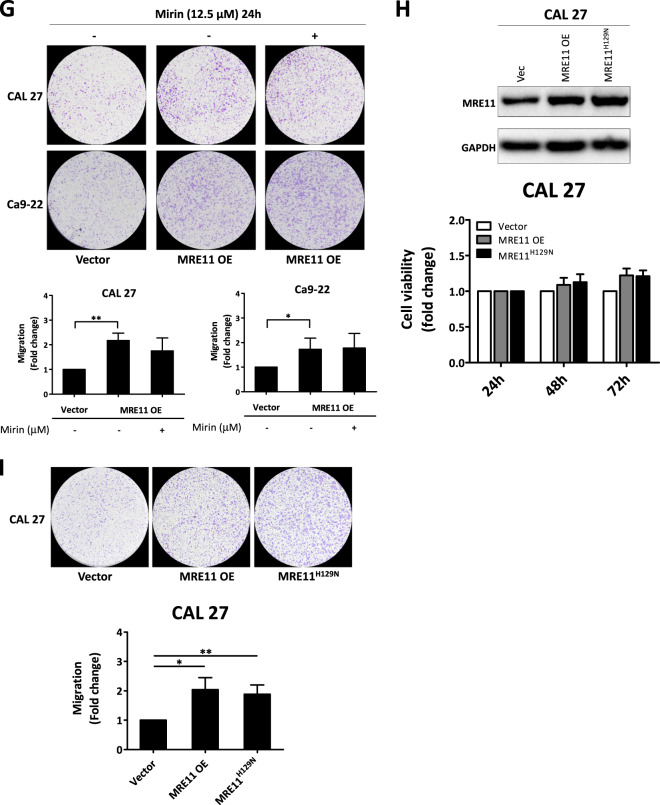

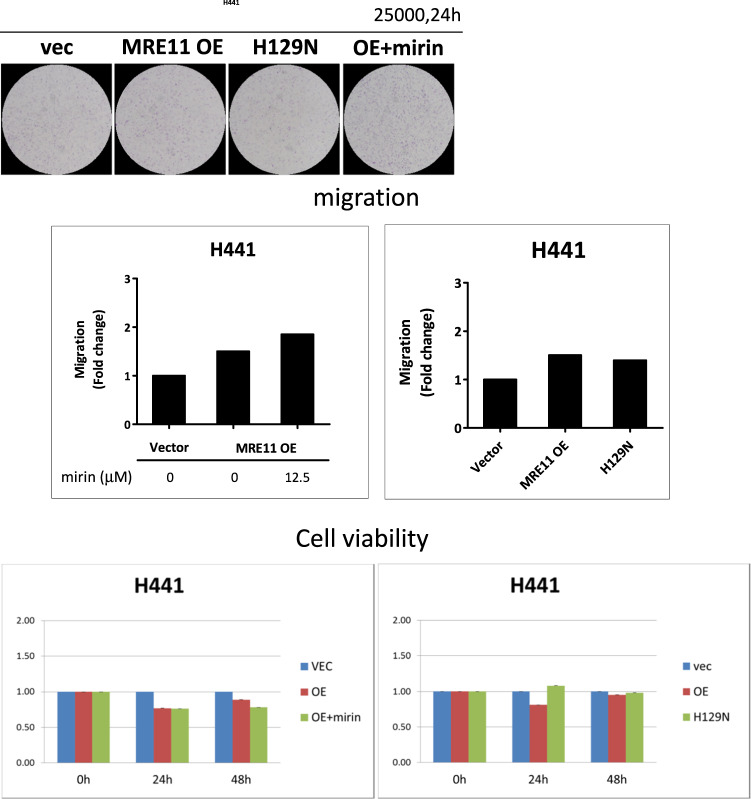
Metastasis-promoting activity of MRE11 in oral cancer cells is independent of its nuclease activity. **A** MRE11 knockdown decreased, while its overexpression increased, wound closure in oral cancer cells. **B** MRE11 knockdown decreased, while its overexpression increased, transwell migration in oral cancer cells. **C** MRE11 knockdown decreased, while its overexpression increased, transwell invasion in oral cancer cells. **D** MRE11 knockdown decreased, while its overexpression increased, epithelial-to-mesenchymal transition in oral cancer cells. **E** Mirin, a MRE11 nuclease inhibitor, increased the expression of γH2AX, an indicator of DSB, in oral cancer cells upon ionizing radiation exposure. **F** Mirin treatment did not inhibit the proliferation-promoting activity of MRE11 in oral cancer cells. **G** Mirin treatment did not inhibit the migration-promoting activity of MRE11 in oral cancer cells. **H** Nuclease-deficient MRE11 with H129N mutation showed proliferation-promoting activity. **I** Nuclease-deficient MRE11 with H129N mutation showed migration-promoting activity.

The effect of MRE11 expression on oral cancer cell metastasis was analyzed in vitro. MRE11 knockdown decreased, while its overexpression increased, oral cancer cell migration as determined by wound healing assay (Fig. 2A). Further transwell migration and invasion assays also showed similar results to the wound healing assay (Fig. 2B, C). We further analyzed the involvement of epithelial-to-mesenchymal transition (EMT) in MRE11-induced oral cancer cell metastasis. Indeed, MRE11 knockdown increased the expression of epithelial markers E-cadherin and ZO-1, whilst decreasing expression of mesenchymal markers vimentin, twist, and β-catenin (Fig. 2D). Conversely, MRE11 overexpression decreased expression of epithelial markers and increased expression of mesenchymal markers (Fig. 2D).

### MRE11 nuclease activity is essential for radioresistance and chemoresistance, but not cancer cell migration, in oral cancer cells

Since MRE11 is a nuclease involved in DSB repair, it is logical to subsequently determine whether the DSB nuclease activity of MRE11 is indispensable to its metastasis-promoting activity. Mirin is a MRE11 nuclease inhibitor and it induced oral cancer cell death at 25 and 50 μM, but not at 12.5 μM, when compared with untreated controls (Fig. S1H) [14]. As depicted in Fig. 2E, mirin decreased nuclease activity of MRE11 and led to increased γH2AX expression upon IR exposure. However, mirin did not inhibit proliferation or migration in oral cancer cells with MRE11 overexpression (Fig. 2F, G). Also, the H129N mutant of MRE11, which is defective in DSB repair, did not inhibit the oral cancer-promoting activities of MRE11 (Fig. 2H, I).

**Fig. 3:**
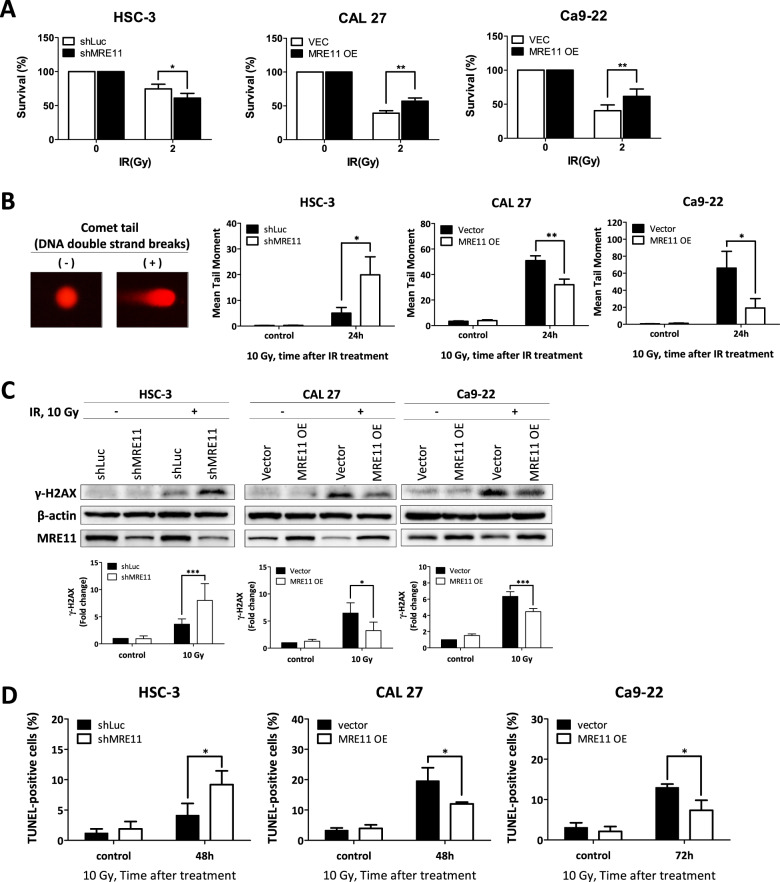

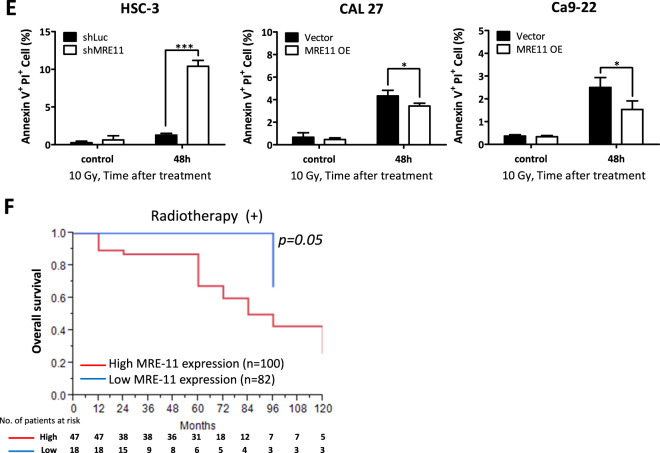
High MRE11 expression in oral cancer cells leads to chemoresistance, radioresistance, and decreased patient survival. **A** The effect of MRE11 knockdown and overexpression on colony formation in oral cancer cells after ionizing radiation exposure. **B** The effect of MRE11 knockdown and overexpression on comet tail formation, an indicator for DSBs, in oral cancer cells after ionizing radiation treatment. **C** The effect of MRE11 knockdown and overexpression on γH2AX expression, an indicator for DSBs, in oral cancer cells after ionizing radiation exposure. **D** The effect of MRE11 knockdown and overexpression on oral cancer cell apoptosis, determined by TUNEL positivity, after ionizing radiation treatment. **E** The effect of MRE11 knockdown and overexpression on oral cancer cell apoptosis, determined by Annexin V positivity, after ionizing radiation treatment. **F** The effect of MRE11 expression, determined by IHC, on overall survival of oral cancer patients after radiotherapy.

Radiotherapy is commonly used for oral cancer treatment due to its ability to cause significant DSBs in cancer cells. Since MRE11 is a DNA double-strand break repair protein, we addressed its effect on oral cancer cell viability upon IR treatment. Knockdown of MRE11 decreased the viability of oral cancer cells upon IR while its overexpression resulted in the opposite effect (Figs. 3A and S2). Furthermore, MRE11 knockdown increased DSBs while its overexpression decreased DSBs upon IR treatment, as determined by neutral comet assay (Fig. 3B). MRE11 knockdown increased the expression of the DSB marker γH2AX, while its overexpression decreased γH2AX expression (Fig. 3C). Further TUNEL study for determination of apoptotic cells also demonstrated that IR led to an increase in TUNEL-positive cells upon MRE11 knockdown, while MRE11 overexpression led to a decrease (Fig. 3D). For analysis of the effect of MRE11 on IR-induced early apoptosis, Annexin V immunofluorescent staining was performed and showed that MRE11 knockdown increased Annexin V-positive cells while its overexpression had the opposite effect (Fig. 3E).

In addition to IR, we also used CDDP, a chemotherapeutic agent frequently used in oral cancer treatment for its ability to cause DNA damage, to study the effect of MRE11 expression on CDDP-induced cancer cell death. MRE11 knockdown led to a decrease in cell viability but an increase in comet tail formation, γH2AX expression, TUNEL-positive cells, and Annexin V-positive cells, upon CDDP treatment (Fig. S3A–E).

### High MRE11 expression in oral cancer tissues was associated with advanced cancer stage, radioresistance, and chemoresistance

The effect of MRE11 expression, as determined by immunohistochemistry, was clinically correlated with the outcomes of radiotherapy and chemotherapy in oral cancer patients. High MRE11 expression in oral cancer tissues was associated with decreased overall survival following radio- or chemotherapy, indicating a positive correlation with radio- (Fig. 3F) and chemoresistance (Fig. S3F).

Since MRE11 expression was associated with resistance to radio- and chemotherapy, we further analyzed the relationship between MRE11 expression and various clinical behaviors. As shown in Table 1, high MRE11 expression in oral cancer tissues was associated with larger tumor size, increased lymph node metastasis, and advanced cancer stage (Table 1). Patients with high MRE11 expression also had a higher likelihood of receiving radiotherapy, but no association was found with alcohol, betel nut chewing, or cigarette smoking (Table 1). We also analyzed the connection between clinicopathological characteristics of OSCC patients and overall survival. Larger tumor size, lymph node metastasis, radiotherapy, and high MRE11 expression in cancer tissues were risk factors for decreased overall survival in oral cancer patients, as determined by univariate cox regression analysis (Table 2). However in multivariable analysis, only larger tumor size, lymph node metastasis, and high MRE11 expression in cancer tissues were significant risk factors (Table 2).

**Table 1:** The association of MRE-11 expression and clinicopathological characteristics of OSCC patients using logistic regression.

Variables	Categories	MRE11		*p* value	Crud OR (95% CI)	Adj OR (95% CI)
		Low	High			
		*N* (%)	*N* (%)			
Histopathological grade	I	72 (44.2)	91 (55.8)	0.86	1	1
	II + III + IV	8 (42.1)	11 (57.9)		1.09 (0.42–2.94)	1.27 (0.47–3.59)
Tumor size	T1	45 (54.9)	37 (45.1)	0.0071	1	1
	T2–T4	35 (35.0)	65 (65.0)		2.26 (1.25–4.14)	2.28 (1.23–4.25)
Lymph node metastasis	No	71 (52.2)	65 (47.8)	<0.0001	1	1
	Yes	9 (19.6)	37 (80.4)		4.49 (2.09–10.57)	4.43 (2.03–10.53)
Pathologic stage	I + II	59 (56.2)	46 (43.8)	<0.0001	1	1
	III + IV	21 (27.3)	56 (72.7)		3.42 (1.84–6.54)	3.39 (1.78–6.63)
Radiotherapy	No	62 (53.0)	55 (47.0)	0.0008	1	1
	Yes	18 (27.7)	47 (72.3)		2.94 (1.55–5.76)	3.39 (1.74–6.87)
Sex	Female	7 (63.6)	4 (36.4)	0.18	1	1
	Male	73 (42.7)	98 (57.3)		2.33 (0.68–9.15)	1.77 (0.46–7.55)
Alcohol drinking	No	21 (45.7)	25 (54.3)	0.63	1	1
	Yes	59 (43.4)	77 (56.6)		1.19 (0.59–2.38)	0.95 (0.43–2.07)
Betel quid chewing	No	17 (43.6)	22 (56.4)	0.86	1	1
	Yes	63 (44.1)	80 (55.9)		1.07 (0.50–2.25)	0.81 (0.34–1.86)
Cigarette smoking	No	18 (52.9)	16 (47.1)	0.14	1	1
	Yes	62 (41.9)	86 (58.1)		1.81 (0.82–4.07)	1.77 (0.21–1.51)

**Table 2:** Association between clinicopathological characteristics of OSCC patients and overall survival.

Variable		*N*	Univariate	*p* value	Multivariable	*p* value
			HR (95% CI)		HR (95% CI)	
Histopathological grade	I	163	1		–	–
	II + III + IV	19	1.11 (0.38–2.61)	0.83	–	–
Tumor size	T1(<2 cm)	82	1	0.0006	1	0.02
	T2–T4	100	3.21 (1.62–6.82)		2.26 (1.12–4.86)	
Lymph node metastasis	No	136	1	<0.0001	1	0.004
	Yes	46	3.78 (1.99–7.20)		2.71 (1.39–5.29)	
MRE11	Low	80	1	0.0002	1	0.01
	High	102	4.23 (1.90–11.234)		2.94 (1.26–8.03)	
Radiotherapy	No	117	1	0.03	1	0.65
	Yes	65	2.61 (1.07–3.81)		1.17 (0.60–2.31)	

### MRE11 activates RUNX2, CXCR4, and AKT, while it inhibits FOXA2, to promote EMT and tumor growth and metastasis in oral cancer

The clinical association of high MRE11 expression with malignant oral cancer behaviors and reduced patient survival prompted us to further explore its role and underlying mechanisms in oral cancer using cell models. Using RT [15] Profiler PCR Array—Human Tumor Metastasis (SABioscience), which evaluates 84 genes involved in different tumor metastasis pathways, we compared differential RNA expression of tumor metastasis-associated genes when MRE11 was overexpressed (Fig. S4A). In MRE11-overexpressing cells, RNA expression of CXCR4, a cell membrane protein involved in cancer cell migration and invasion [12], was increased (Fig. S4A), but this was reversed when cells were cotreated with siCXCR4 (Fig. S4B).

**Fig. 4:**
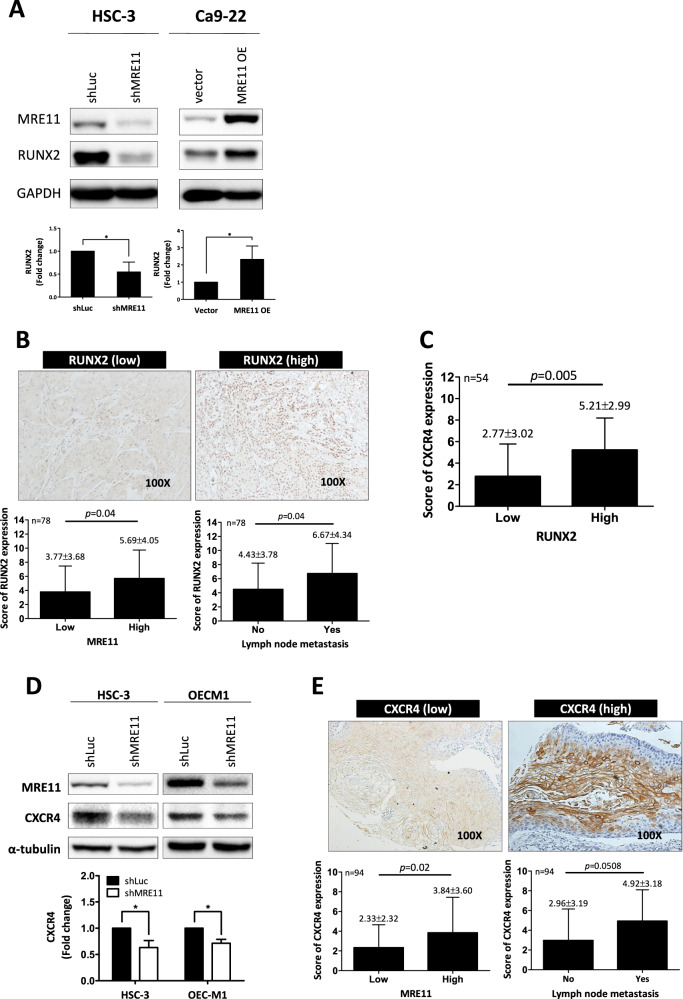

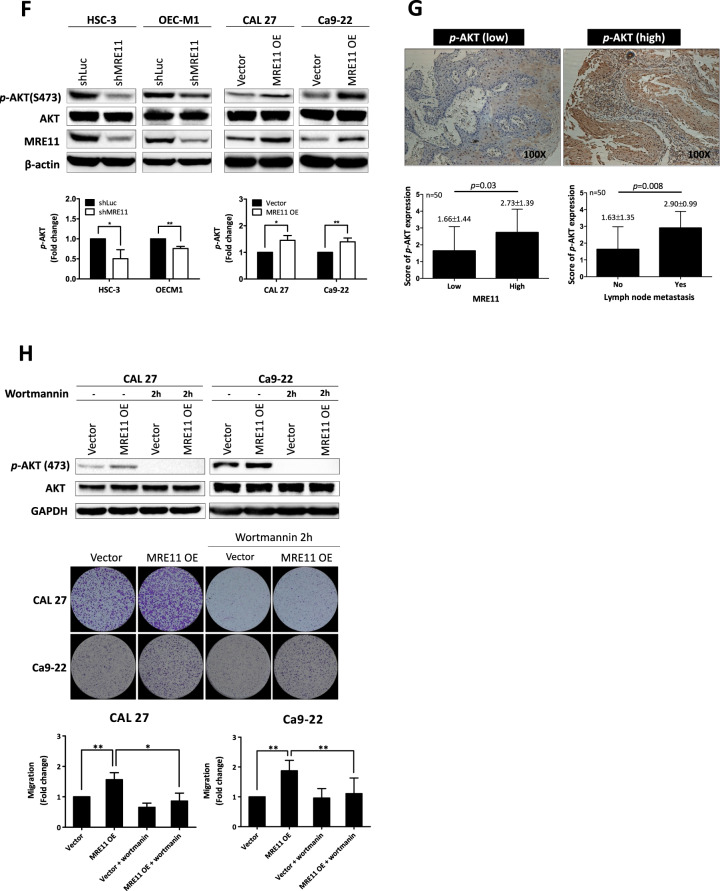

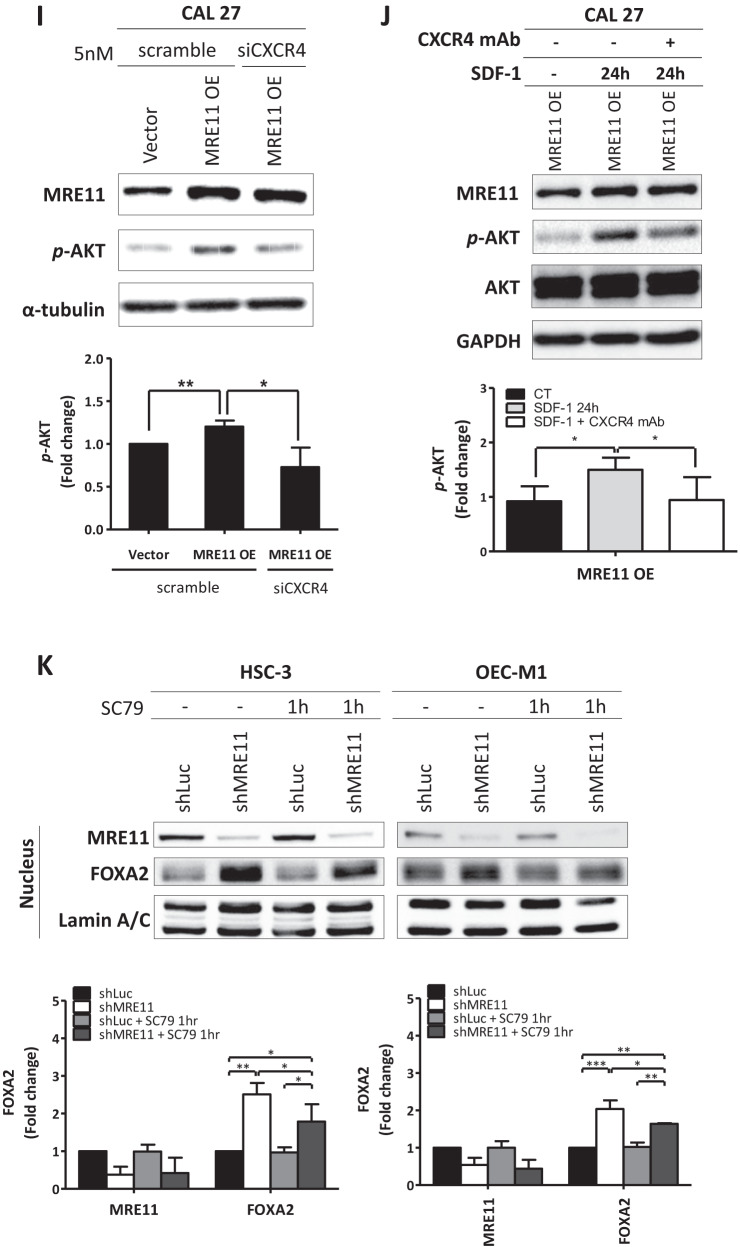

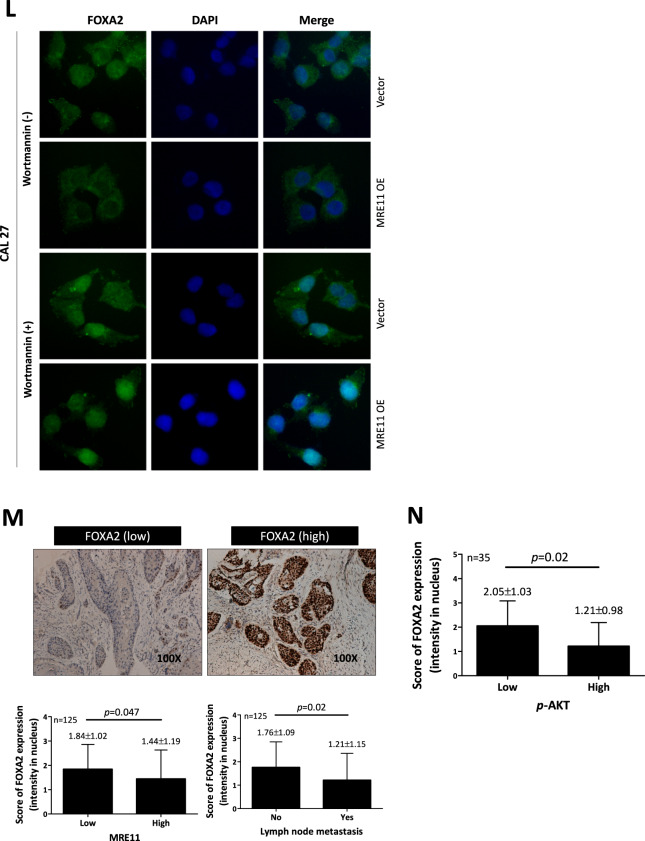
CXCR4, RUNX2, AKT, and FOXA2 are involved in MRE11-induced oral cancer metastasis. **A** MRE11 knockdown led to decreased RUNX2 expression, while MRE11 overexpression led to increased RUNX2 expression, in oral cancer cells. **B** The correlation of RUNX2 expression in oral cancer tissues with MRE11 expression and lymph node metastasis in oral cancer patients. **C** A positive correlation between RUNX2 and CXCR4 expression in oral cancer tissues. **D** CXCR4 expression in oral cancer cells was decreased when MRE11 was knockdowned. **E** MRE11 and CXCR4 were positively correlated in primary oral cancer tissues and CXCR4 was more highly expressed in oral cancer tissues with lymph node metastasis. **F** MRE11 knockdown led to decreased pAKT expression, while MRE11 overexpression led to increased pAKT expression, in oral cancer cells. **G** The correlation between MRE11 and pAKT expression in oral cancer tissues. **H** Wortmannin, a PI3K/AKT inhibitor, reversed the increased oral cancer migration induced by MRE11 overexpression. **I** CXCR4 silencing reversed the increased pAKT expression caused by MRE11 overexpression. **J** CXCR4 blocking mAb reversed the increased pAKT expression caused by SDF-1, a CXCR4 activator, in MRE11-overexpressing oral cancer cells. **K** MRE11 knockdown in oral cancer cells led to increased expression of nuclear FOXA2, which was partially reversed by cotreatment with AKT activator SC79. **L** MRE11 overexpression in oral cancer cells decreased the nuclear expression of FOXA2 while treatment with wortmannin reversed the increased nuclear expression of FOXA2 induced by MRE11 overexpression. **M** Correlation of MRE11 and FOXA2 expression in oral cancer tissues and metastatic lymph nodes. **N** Correlation of phospho-AKT and FOXA2 expression in oral cancer tissues and metastatic lymph nodes.

RUNX2 is a transcription factor that promotes CXCR4 expression [16, 17]. Further immunoblotting analysis showed that RUNX2 expression was upregulated when MRE11 was overexpressed, while its expression was downregulated when MRE11 was knockdowned (Fig. 4A). Immunohistochemistry analysis also showed a positive correlation between the expression of MRE11 and RUNX2 in oral cancer tissues (Fig. 4B).

A positive correlation between the expression of RUNX2 and CXCR4, as determined by immunohistochemistry analysis, was observed in oral cancer tissues (Fig. 4C). Further western blotting analysis confirmed that CXCR4 expression in oral cancer cells was decreased when MRE11 was knockdowned (Fig. 4D). We then checked the association between MRE11 and CXCR4 by immunohistochemistry using oral cancer tissue specimens. As shown in Fig. 4E, MRE11 and CXCR4 were positively correlated in primary oral cancer tissues and CXCR4 was more highly expressed in oral cancer tissues with lymph node metastasis.

Previous studies have reported that CXCR4 signaling is involved in the establishment of lymph node metastasis in oral cancer through AKT activation [18]. In this study, S473 phosphorylation of AKT was decreased upon MRE11 knockdown but was increased upon MRE11 overexpression in oral cancer cells (Fig. 4F). Further study using clinical oral cancer specimens confirmed a positive correlation between the expression of MRE11 and S473 phospho-AKT (Fig. 4G). To determine whether AKT activation is indeed the downstream effector of MRE11, MRE11-overexpressed cells were treated with wortmannin, a PI3K/AKT inhibitor, and the result showed that MRE11-induced cancer cell migration was indeed blocked by cotreatment with wortmannin (Fig. 4H). CXCR4 knockdown also reversed AKT phosphorylation in oral cancer cells induced by MRE11 overexpression (Fig. 4I). In addition, AKT phosphorylation induced by SDF, a CXCR4 ligand, in MRE11-overexpressing cells was reversed by cotreatment with CXCR4 mAb (Fig. 4J).

Transcription factor FOXA2, a member of the forkhead box protein 2A/winged-helix family, binds to the promoter of CDH1 and upregulates the expression of E-cadherin, the gene product of CDH1 [19]. A previous study has demonstrated that FOXA2 is hypermethylated in various cancer cell lines [20]. To explore the underlying mechanisms of MRE11-induced oral cancer cell metastasis, the expression of FOXA2 was analyzed. MRE11 knockdown in oral cancer cells led to increased expression of FOXA2, which was blocked by cotreatment with SC79, an AKT activator (Fig. 4K). FOXA2 is a transcriptional factor which, when activated, moves to the nucleus to activate the transcription of downstream effectors including E-cadherin [21]. While FOXA2 stayed in the cytosol when MRE11 was overexpressed, it moved to the nucleus when MRE11-overexpressing cells were cotreated with wortmannin (Fig. 4L). However in MRE11-knockdowned cells, FOXA2 moved to the nucleus (Fig. S5A). We also observed a negative correlation between the expression of MRE11 and FOXA2 in both oral cancer tissues and metastatic lymph nodes (Fig. 4M), and a negative correlation between the expression of S473 phospho-AKT and FOXA2 (Fig. 4N). A negative correlation between MRE11 and E-cadherin expression and a positive correlation between FOXA2 and E-cadherin (a downstream effector of FOXA2) expression were also observed in oral cancer tissues (Fig. S5B, C). Furthermore, a positive correlation between MRE11 and CXCR4 expression and a negative correlation between both MRE11/FOXA2 expression and CXCR4/FOXA2 expression were observed from heat map view and scatter/correlation plot using oral cancer microarray datasets from ONCOMINE Cancer Profiling Database (https://www.oncomine.org) (Fig. S5D).

### MRE11-promotion of tumor growth and metastasis was suppressed by inhibition of CXCR4 activity

**Fig. 5:**
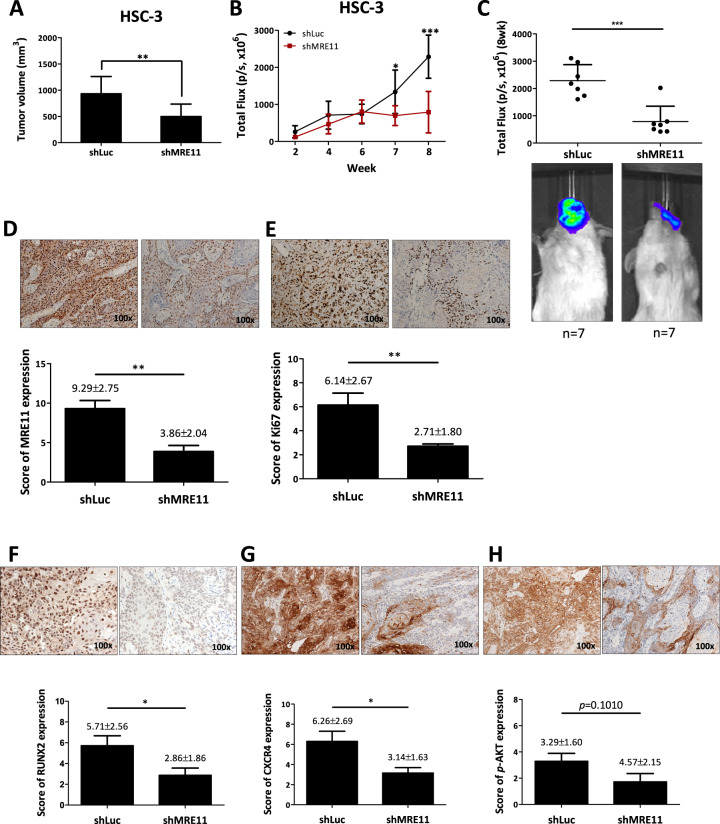

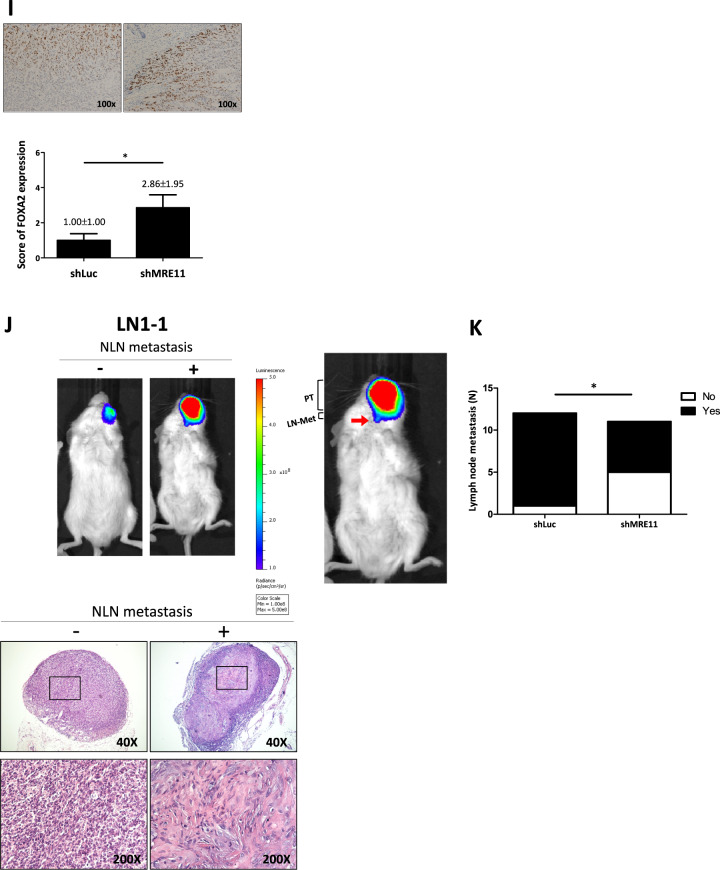
MRE11 knockdown decreased orthotopic oral tumor growth and cervical lymph node (CLN) metastasis with decreased expression of CXCR4 and pAKT but increased expression of FOXA2 in oral cancer tissues. **A** The effect of MRE11 knockdown in oral cancer cells on orthotopic oral tumor volume. **B** The effect of MRE11 knockdown in oral cancer cells on weekly total flux of orthotopic oral tumors. **C** The effect of MRE11 knockdown in oral cancer cells on total flux of orthotopic oral tumors at sacrifice. **D**–**I** The effect of MRE11 knockdown in oral cancer cells on the expression of MRE11, Ki67, RUNX2, CXCR4, pAKT, and FOXA2 in oral tumor tissues. **J** An example of CLN metastasis shown by IVIS imaging and H&E stain. **K** Quantitative data of CLN metastasis in mice when MRE11 expression in oral cancer cells was knockdowned.

Finally, the effect of MRE11 on oral cancer behaviors was addressed with animal models. An orthotopic HSC3 oral cancer model was developed in SCID mice by injecting cancer cells into the buccal area of mice. Smaller tumor volume was observed in MRE11 knockdown group (shMRE11) compared to control group (shLuc) (Fig. 5A). Tumor lesions were evaluated weekly with IVIS analysis, and a significantly lower luciferase activity determined by total flux was observed at 7th and 8th weeks in MRE11 knockdown group (shMRE11) compared to control group (shLuc) (Fig. 5B). The luciferase activity was significantly lower in MRE11 knockdown group at sacrifice (Fig. 5C). The oral tumors were collected for immunohistochemistry analysis for the expression of MRE11, Ki67, RUNX2, CXCR4, phospho-AKT, and FOXA2. In agreement with the in vitro and clinical data, knockdown of MRE11 led to decreased expression of Ki67, CXCR4, and phospho-AKT, but increased expression of FOXA2 (Fig. 5D–I). We also analyzed cervical lymph node (CLN) metastasis in the orthotopic mouse model by using LN1-1 oral cancer cells, a subline of OEC-M1 isolated from metastatic CLNs. Figure 5J shows an example of CLN metastasis demonstrated by IVIS imaging and Fig. 5K shows H&E stains. The quantitative data of CLN metastasis showed decreased NLN metastasis in mice injected with oral cancer cells with MRE11 knockdown. Of note, MRE11 knockdown in LN1-1 cells decreased cell viability at 72 h (Fig. S6A) and decreased transwell migration at 24 h after incubation (Fig. S6B). In orthotopic mouse model, MRE11 knockdown in LN1-1 cells led to decreased total flux (Fig. S6C) and reduced tumor volume (Fig. S6D) of orthotopic oral tumors. We also noticed decreased expression of mesenchymal marker vimentin and increased expression of epithelial marker E-cadherin in the above mentioned orthotopic tumor model (Fig. S7A, B).

**Fig. 6:**
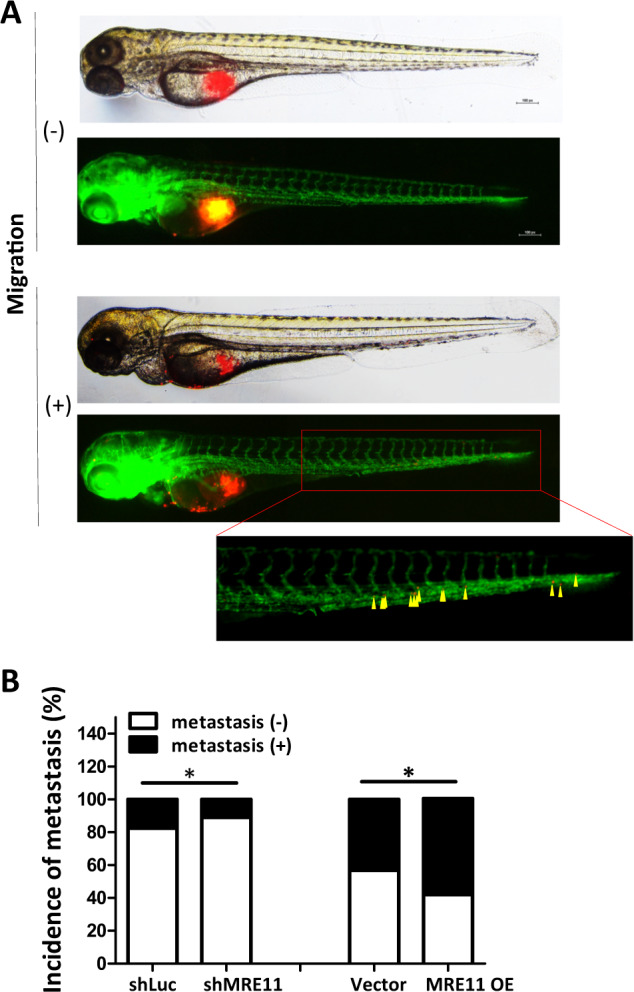

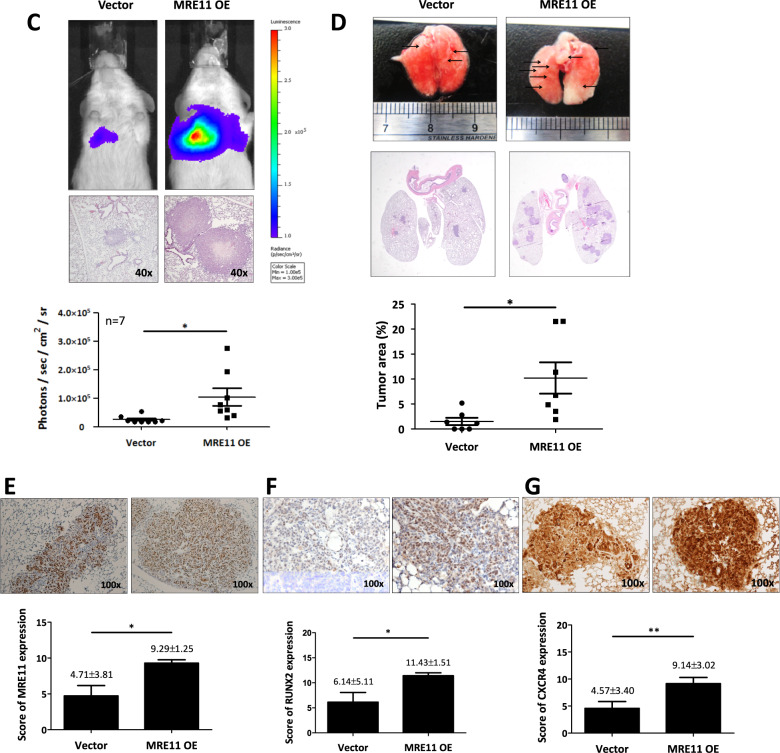

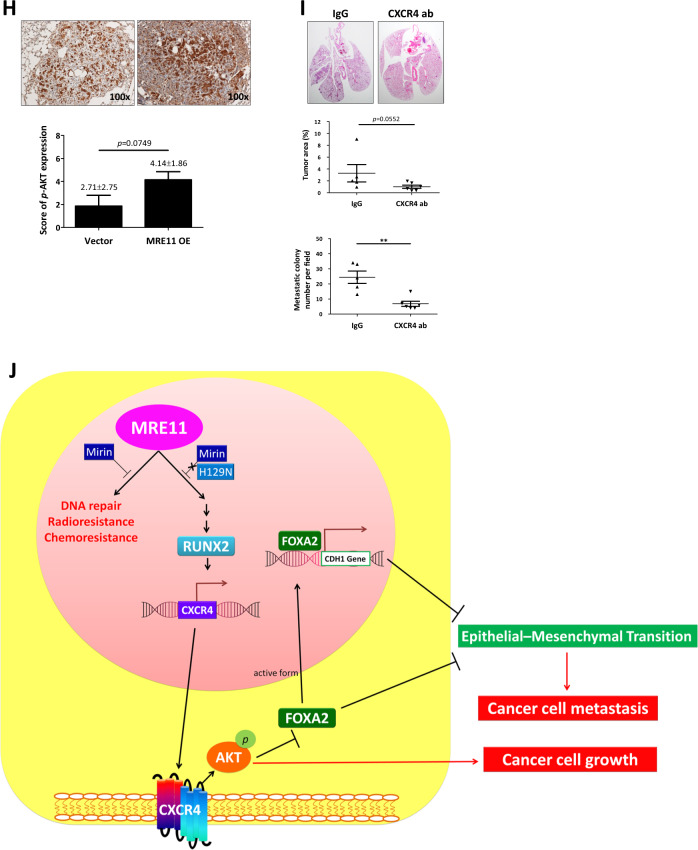
MRE11 expression in oral cancer cells is associated with metastasis in zebrafish and mouse models. **A** An example of oral cancer cell migration in zebrafish. **B** Quantitation of the effects of MRE11 knockdown and overexpression in oral cancer cells on migration in zebrafish model. **C** The effect of MRE11 overexpression in oral cancer cells on total flux of lung metastasis. **D** The effect of MRE11 overexpression in oral cancer cells on metastatic lung tumor area. **E**–**H** The effect of MRE11 overexpression in oral cancer cells on the expression of MRE11, RUNX2, CXCR4, and pAKT in metastatic lung tumor tissues. **I** CXCR4 neutralizing antibody reverse the cancer metastasis promoted by MRE11 overexpression in mouse model. Representative microscopic views of the lung sections. Hematoxylin-positive blue-colored nodules indicate metastatic colony number (Student’s *t* test) and tumor area (%) (Mann–Whitney *U* test). **J** Schematic diagram for MRE11 signaling pathway and activity.

To study the effect of MRE11 expression on cancer cell metastasis in vivo, we injected the oral cancer cells into the perivitelline space of 2 days old zebrafish embryos. MRE11-knockdowned oral cancer cells showed decreased migration into GFP-stained blood vessels, and MRE11-overexpressed oral cancer cells showed increased migration (Fig. 6A, B and Table S2). Using SCID mice tail vein injection model, we observed increased lung metastasis, evidenced by increased luminescence and tumor nodule formation, in mice injected with MRE11-overexpressing oral cancer cells (Fig. 6C, D). Furthermore, decreased FOXA2 expression and elevated expression of MRE11, Ki67, CXCR4, and S473-phospho-AKT were observed in MRE11-overexpressing lung tumor nodules (Fig. 6E–H).

To explore potential treatment strategies for MRE11-overexpressing oral cancers in vivo, we tested the efficacy of targeting CXCR4 with neutralizing antibody in mice (Fig. 6I). Neutralizing CXCR4 antibody mitigated lung metastasis promotion by MRE11 overexpression, as evidenced by decreased luminescence and tumor nodule formation.

## Discussion

This is the first study to highlight the significance of MRE11 in oral cancer progression, and adds to the growing body of literature indicating the importance of the chemokine receptor CXCR4 in a multitude of cancers. We have shown that in addition to its well recognized nuclease activity, MRE11 promotes EMT and cancer stemness through nuclease independent activation of RUNX2, CXCR4, and AKT to inhibit FOXA2/E-cadherin, leading to cancer growth and metastasis, radio- and chemoresistance, and poor survival in oral cancer patients (Fig. 6J). This pathway provides a range of potential downstream therapeutic targets—in particular chemokine receptor CXCR4—for which real-time clinical assessment with CXCR4 directed PET imaging has recently been developed [22].

### Differential MRE11 nuclease and non-nuclease dependent effects in oral cancer

Although elevated MRE11 expression was associated with a more malignant cancer phenotype, disparate MRE11 nuclease and non-nuclease activities were found to mediate distinct aspects of the cancer phenotype. MRE11 nuclease dependent activity appears to be pivotal in clastogenic resistance to chemo and radiotherapy, with specific MRE11 nuclease inhibitor (Mirin) and MRE11 nuclease-deficient mutant (H129N) resulting in increased sensitivity to clastogenic therapies. Consequently it appears that MRE11 nuclease activity, although protective of genomic integrity in the normal cell phenotype, may be maladaptive in the cancer phenotype by permitting tumor cell viability in the face of exogenous DNA damage from clastogenic therapies and endogenous DNA replication stress. Strikingly, inhibition of MRE11 nuclease activity had no effect on tumor proliferation and metastasis, with our data indicating that MRE11 may separately mediate these effects via a nuclease independent pathway involving RUNX2—a transcription factor which promotes CXCR4 expression, AKT activation, and subsequent inhibition of FOXA2/E-Cadherin activity. Although these nuclease dependent and independent activities appear to be responsible for disparate cancer behaviors, mutual regulation may exist between these pathways with RUNX2 deficiency previously shown to result in loss of MRE11/RAD50/NBS1 DNA repair complex, indicating that RUNX2 may be an upstream regulator of MRE11 nuclease activity [23]. Furthermore, AKT may inhibit DSB repair in colon cancer cells via inhibition of MRE11 by p70S6 kinase [24], suggesting the presence of a feedback loop between MRE11 and AKT [25]. This feedback loop is not alone and has also been reported in other couples, e.g., ZEB/miR-200, MDM2/p53, PI3K/mTOR, and E2F1-C/EBPα [15, 26–29]. Mutual regulation of MRE11 is therefore multifactorial and incompletely elucidated, and may involve RUNX2 and AKT in a cell type-dependent manner.

The nuclease-independent role of MRE11 in promotion of EMT and metastasis in oral cancer cells was also tested and confirmed in lung cancer cells (data not shown). Whether this mechanism is unique to oral and lung cancer cells that we tested or is universal to various cancer cell types merits further investigation.

### MRE11 and CXCR4 signaling is not conserved across different cell types

The MRE11 signaling pathway is not conserved across different cell types, with our prior report indicating that MRE11 mediates its effects in breast cancer via STAT3 and its downstream effectors cyclin D, Myc, and BCL-xL [11]. In the current study, we did not observe similar involvement of STAT3 in oral cancer (Fig. S8), suggesting that cell type differences between oral cancer (squamous cell carcinoma) and breast cancer (adenocarcinoma) may contribute to differences in the MRE11-preferential signaling pathways. Likewise, the chemokine receptor CXCR4 has multiple upstream mediators, with upregulation in renal cell carcinoma described in response to hypoxia inducible factor (HIF1a) [30] and galectin 1 [31], and in breast cancer with angiotensin II type I receptor (AGTR-1) and NF kappa B [32, 33].

### Future clinical applications targeting CXCR4 for treatment of MRE11-overexpressing oral cancer

Our findings suggest that MRE11 inhibition may pose an attractive therapeutic target in oral squamous cell cancer, but potential problems exist in balancing the complexity of altering a potentially beneficial (preservation of genome integrity in the normal phenotype) and potentially maladaptive response (abrogation of the DDR to clastogenic therapies and endogenous tumor replication stress), together with the current paucity of suitable clinical antagonists, real-time functional DDR assays and noninvasive methods of MRE11 quantification. Nonetheless, DDR inhibitors present the next generation of anticancer therapeutic strategies, with the PARP inhibitors in BRCA-mutant ovarian and breast cancers the paradigm in this field [34]. In this regard, tumors expressing phosphomimetic (inactive) MRE11 are more sensitive to the PARP inhibitor olaparib, compared with those expressing unphosphorylatable MRE11, suggesting that patients with elevated Plk1 expression may benefit from olaparib treatment [35]. CXCR4 may currently provide a more mature therapeutic target than MRE11. CXCR4 has been shown in this study to be critical to OSCC invasion and metastasis, and has garnered attention in recent years for its role in multiple other tumor types, with overexpression in at least 23 other cancer types [36]. The development of specific antagonists such as balixafortide, which has recently completed Phase I clinical trials in HER2 negative metastatic breast cancer, shows promise [37]. Together with ^68^Ga-pentixafor/PET imaging, this may allow the noninvasive real-time assessment of CXCR4 specific therapies in the future [22].

### Upstream regulators for MRE11 and the role of replication stress

Upstream regulation and overexpression of MRE11 in oral cancer remains one of the key issues to be resolved. Whilst this study focused on the downstream events of MRE11 in oral cancer, we have shown that MRE11 overexpression is not simply reactive to replication stress, but is likely to be a complex and multifactorial process comprising epigenetic regulation, mutual regulation, and other upstream regulators. Cancer cells are constantly exposed to replication stress and replication-associated DDR, which may in turn activate MRE11 expression [10, 38], but we found no association between MRE11 expression and the expression of phosphorylated ATM, phosphorylated ATR, and γ-H2AX in oral cancer tissues (Fig. S9). Instead, our results point towards epigenetic regulation playing a more prominent role in MRE11 overexpression, with data showing a significant negative correlation with DNA methylation at the putative promoter region (CpG site cg26262057), regardless of HPV status. The upstream regulation of MRE11 may also involve mutual regulation by AKT/MRE11 and RUNX2/MRE11 as discussed previously, with other proposed factors including ribosomal s6 kinase (Rsk), Polo-like kinase 1 (Plk1), and FGFR2. Rsk and Plk1 both suppress DNA-damage checkpoint signaling by phosphorylating and inhibiting MRE11 activity [35, 39], whilst FGFR2 regulates MRE11 expression through the MEK/ERK/POU1F1 pathway in breast cancer [40].

## Conclusions

In this study, we conclude that MRE11 may serve as a crucial prognostic factor and therapeutic target in oral cancer, displaying dual nuclease dependent and independent roles that permit separate targeting of tumor vulnerabilities to the DNA damage response and EMT, migration and metastasis. CXCR4, a downstream effector of RUNX2 transcription factor, presents an exciting and viable target in oral cancer, with both antagonists and CXCR4 directed imaging in the mature stage of development.

## Materials and methods

Details of all methods are found in Supplementary Information.

## Supplementary information

Supplementary information

Supplementary data
