# Supplementary material for: MRE11 promotes oral cancer progression through RUNX2/CXCR4/AKT/FOXA2 signaling in a nuclease-independent manner
Source: Oncogene. 2021 Apr 29;40(20):3510–32. doi: 10.1038/s41388-021-01698-5 (PMC8134045; doi:10.1038/s41388-021-01698-5)
Supplement: Supplementary file 1 — Supplementary information [file 41388_2021_1698_MOESM1_ESM.pdf]

Supplementary information

Materials and Methods

TCGA data

Gene expression data (normalized count of RNA-seq data) and DNA methylation data (β value of methylation array data) of head and neck squamous cell carcinoma in TCGA (TCGA-HNSC) were downloaded from the TCGA website (https://cancergenome.nih.gov/). Pearson correlation analysis was applied to calculate the correlation between gene expression and DNA methylation data.

Patient samples

Patients with OSCC were recruited from the Department of Oral and Maxillofacial Surgery, Kaohsiung Medical University Hospital. They were independently diagnosed by two dental pathologists, and the diagnoses were confirmed using clinical and histological data. Each patient's clinical cancer [TNM (tumor–node–metastasis)] stage was determined according to the 1992 criteria of the American Joint Cancer Committee/Union International Cancer Control (AJCC/UICC) [8]. Overall survival (OS) was defined as the interval between the date of diagnosis and death. Disease-free survival (DFS) was defined as the interval from the date of diagnosis and date of first recurrence, locoregional or systemic. All other events were censored. IRB approval and informed consent from patients were obtained (KMUH-IRB-20130300).

Tissue microarray and immunohistochemical analysis

To determine the immunohistochemical expression of MRE11 in OSCC, FFPE (formalin-fixed, paraffin-embedded) tumor tissues were examined in a tissue microarray. Slides from hematoxylin-eosin–stained sections were reviewed by a specialist pathologist to select representative areas of tumor and normal tissues for core biopsy within the primary paraffin blocks. Tissue microarray was constructed following standard tissue array–producing protocols [5]. Four-micrometer sections were cut from recipient tissue microarray blocks by an adhesive-coated tape sectioning microtome (Alphelys). Immunohistochemical analysis was performed using an automatic IHC-staining device, according to the manufacturer's instructions (Bond-max Automated Immunostainer; Vision Biosystems, Melbourne, Australia) [1]. Goat anti-human MRE11 polyclonal antibody (dilution 1:200) was purchased from Santa Cruz Biotechnology (Santa Cruz, CA), and the same antibody was also used for immunoblotting. Images of immunohistochemically stained sections were captured by Nikon E600 microscope (Nikon, Tokyo, Japan), and processed with Adobe Photoshop 6.0 (Adobe System Incorporated, San Jose, CA). Only nuclear staining of MRE11 was counted.

The percentage of positively-stained tumor cells was determined semiquantitatively by assessing tumor sections, with each sample assigned to one of the following categories: 0 (0-4%), 1 (5-24%), 2 (25-49%), 3 (50-74%), or 4 (75-100%). Additionally, intensity

of immunostaining was determined as 0 (negative), 1 (weak), 2 (moderate) or 3 (strong). The total score was designated as the percentage of positively-stained cells multiplied by the weighted intensity of staining for each sample. For further statistical analysis, scores ≤ 9 were categorized as low expression, and >9 were categorized as high expression (cut point at median). The staining was determined separately for each specimen by two independent experts simultaneously under the same conditions [2, 7].

Oncomine™ platform

Oncomine™ , an integrated cancer microarray database and web-based data-mining platform [6], was used to search for genes that may be correlated with MRE11.

Cell culture

Human oral squamous cancer cell lines Ca9-22, CAL 27, HSC-3, OEC-M1 and LN1-1, a subline of OEC-M1 isolated from a metastatic cervical lymph node [9], were obtained and authenticated (genotype and phenotype authentication) by the Bioresource Collection and Research Center, Taiwan (www.bcrc.firdi.org.tw). All media were purchased from Thermo Fisher Scientific (Waltham, MA, USA) and supplemented with 10% (vol/vol) fetal bovine serum (FBS) (Biological Industries, Haemek, Israel) and antibiotics (100 units/mL penicillin, 100 μg/mL streptomycin, and 2.5 μg/mL amphotericin B) (Biological Industries, Haemek, Israel). All cells were grown and

maintained at 37°C in a 5% CO_2 incubator (Thermo Fisher Scientific, Waltham, MA, USA). Cells were regularly tested for mycoplasma contamination.

Virus infection for MRE11 knockdown/overexpression

MRE11 was silenced in oral squamous cell carcinoma cells with a lentivirus carrying a pLKO.1_puro lentiviral vector expressing double-stranded shRNA oligonucleotides targeting the sequence of human MRE11 (shRNA1, 5’-GCTGCGTATTAAAGGGAGGAA-3’, National RNAi Core Facility, Academia Sinica, Taiwan). Another pLKO.1_puro lentiviral vector expressing shRNA targeting firefly luciferase, unrelated to the human genome sequence, was used as negative control (National RNAi Core Facility, Academia Sinica, Taiwan).

For overexpression of MRE11 in OSCC cells, ready-to-use lentiviral particles containing pReceiver Lv105 lentiviral vector which expressed human MRE11 gene were purchased from Genecopoeia (Rockville, MD). For negative control, lentiviral particles which carried an empty pReceiver Lv105 lentiviral vector were used (Genecopoeia, Rockville, MD). Lentiviral infection was performed by adding viral solution to cells, with culture media containing 8 μg/mL polybrene. After infection for 48 hrs, 2μg/mL puromycin was added for selection. Surviving cells were maintained continuously with 2μg/mL puromycin and cultured for further experiments.

XTT cell proliferation assay

XTT (tetrazolium salt 2,3-bis[2-methyloxy-4-nitro-5-sulfophenyl]-2H-tetrazolium-5-carboxanilide) assay was performed to evaluate the effect of MRE11 on OSCC cell proliferation. HSC-3 and OECM1 cells with MRE11 knockdown and Ca9-22 and CAL 27 cells with MRE11 overexpression were seeded (2×10^3 cells per well) in 96-well culture plates. At time points of 24hr, 48hr and 72hr after seeding, the medium was removed, and 150μL of XTT solution (50μg XTT and 0.4μg PMS in 150μL of culture media) (Sigma-Aldrich, St. Louis, MO) was added and incubated at 37°C for 2hr. The assay was performed by measuring optical density (OD) at 475 nm and subtracting nonspecific background at 660 nm (OD_{475nm} - OD_{660nm}).

Immunoblotting analysis

Immunoblotting was performed as described previously [10]. The chemiluminescent signal was captured by Gel Doc™ XR+ Gel Documentation System (BIO-RAD, Hercules, CA). The following antibodies were used: goat anti-human MRE11 (sc-5859, Santa Cruz, CA), rabbit anti-human γ-H2AX (GTX61796, GeneTex, Irvine, CA), rabbit anti-human p-Akt Ser473 (#3787, Cell signaling, Danvers, MA), rabbit anti-human p-Erk (GTX61126, GeneTex, Irvine, CA), rabbit anti-human Akt (#4691, Cell signaling, Danvers, MA), rabbit anti-human Erk (GTX82556, GeneTex, Irvine, CA), rabbit anti-human p-STAT3 (GTX61050, GeneTex, Irvine, CA), rabbit anti-human STAT3 (GTX15523, GeneTex, Irvine, CA), rabbit anti-human ZO-1 (GTX108613, GeneTex,

Irvine, CA), rabbit anti-human E-cadherin (GTX100443, GeneTex, Irvine, CA), rabbit anti-human twist (GTX127310, GeneTex, Irvine, CA), rabbit anti-human vimentin (GTX100619, GeneTex, Irvine, CA), rabbit anti-human β-catenin (ab16051, Abcam, Cambridge, UK), mouse anti-human CD44, rabbit anti-human CXCR-4 (sc-9046, Santa Cruz, CA), rabbit anti-human FOXA2 (ab108422, Abcam, Cambridge, UK), rabbit anti-human Lamin A/C (GTX62457, GeneTex, Irvine, CA), rabbit anti-human GAPDH (GTX100118, GeneTex, Irvine, CA), rabbit anti-human α-tubulin (GTX112141, GeneTex, Irvine, CA), and mouse anti-human β-actin (A5441, Sigma-Aldrich, St. Louis, MO). HRP-conjugated goat anti-rabbit and anti-mouse antibodies were obtained from GeneTex (Irvin, CA).

***In vitro* migration and invasion assays**

Cell migration assay was carried out using Transwell (Corning Costar Corp., Cambridge, MA, USA) membrane filter inserts in 24-well tissue culture plates (6.5mm diameter, 8μm pore size). For invasion assay, Transwell inserts were coated with Matrigel on the upper side. After overexpression or knockdown of MRE11, oral cancer cells were trypsinized and suspended in serum-free medium, and seeded on the upper chamber of transwell filters. Serum-containing medium was added to the lower chamber and incubated for 24hrs at 37°C. The cells were then fixed with 4% formaldehyde, and stained with crystal violet. Non-migrating cells were removed by

wiping the upper side of the filter. The number of migrated cells was determined by Image J software.

Annexin V staining

FITC Annexin V Apoptosis Detection Kit I (BD Biosciences, San Jose, CA) was used to detect apoptotic cells during apoptosis progression after radiation or CDDP treatment. HSC-3 cells with MRE11 knockdown or Ca9-22 and CAL 27 cells with MRE11 overexpression were seeded on 6cm plates. At time points of 24hr and 48hr after radiation or CDDP treatment, 1 x 10^6 cells were collected and washed twice with ice-cold phosphate-buffered saline (PBS; 137 mM NaCl, 2.7 mM KCl, 10 mM Na_2HPO_4, and 2 mM KH_2PO_4 [pH=7.4]). The cells were then stained with Annexin V working solution (5μL of Annexin V-FITC reagent and 5μL of propidium iodide (PI) solution in 100μL of 1X Annexin V binding buffer) for 15 minutes at room temperature. Subsequent analysis was performed with Cytomics FC 500 flow cytometer (Beckman Coulter, Brea, CA, USA). The fluorescence signals of Annexin V-FITC and PI were detected by FL-1 and FL-3 channels respectively, and 10,000 cells were analyzed in 3 independent experiments.

TUNEL staining

APO-DIRECT™ Kit (BD Biosciences, San Jose, CA) was used to detect late apoptotic cells after radiation treatment. HSC-3 cells with MRE11 knockdown or Ca9-22 and

CAL 27 cells with MRE11 overexpression were seeded on 6cm plates. At time points of 24hr and 48hr after radiation treatment, 1 x 10^6 cells were collected and fixed with fix solution (1% formaldehyde in 70% ethanol) for 24hrs at -20°C. The following procedures were performed according to the manufacturer's instructions. Subsequent analysis was performed with Cytomics FC 500 flow cytometer (Beckman Coulter, Brea, CA, USA). The fluorescence signals of FITC-dUTP and PI were detected by FL-1 and FL-3 channels respectively, and 10,000 cells were analyzed in 3 independent experiments.

Ionizing radiation treatment of OSCC cells

HSC-3 cells with MRE11 knockdown or Ca9-22 and CAL 27 cells with MRE11 overexpression were irradiated at doses of 2, 5 and 10 Gy at room temperature using a 6MV linear accelerator (ELEKTA-Sli, Norcross, GA, USA) at the Department of Radiation Oncology, KMU Hospital. The cells were then obtained for further experiments.

γ-H2AX activation after IR exposure

HSC-3 cells with MRE11 knockdown or Ca9-22 and CAL 27 cells with MRE11 overexpression were grown on 6cm plates. After 10 Gy γ-irradiation, the medium was removed and the cells were collected for further protein extraction.

Neutral comet assay

Neutral comet single-cell gel electrophoresis was used for determining irradiation-induced DSBs in HSC-3 cells with MRE11 knockdown or Ca9-22 and CAL 27 cells with MRE11 overexpression. Cells were grown on 6cm-plates and after 10 Gy IR exposure, cells were collected at time points of 24, 48 and 72hr for further comet assay. According to the manufacturer's protocol (Trevigen, Gaithersburg, MD), collected cells (10,000 cells) were mixed with 50μL of low-melting point agarose gel and placed on a comet slide (Trevigen, Gaithersburg, MD), followed by electrophoresis at 21 volts for 20 min in neutral electrophoresis buffer. The slide was washed with ddH_2O for 5 min, DNA was stained with propidium iodide (PI) solution for 5min, followed by a further wash with ddH_2O for 5 min. Comet tails produced by DNA damage in individual cells were visualized with fluorescence microscopy and further quantified with Image J software. Tail moments [(tail mean – head mean) × (% tail DNA/100)] were automatically calculated by software. Three independent experiments were performed and more than 50 individual cells were quantified per data point.

Colony forming assay

HSC-3 cells with MRE11 knockdown or Ca9-22 and CAL 27 cells with MRE11 overexpression were seeded in 6-well plates (1,000 cells/well), exposed to 2, 5, 10 Gy irradiation and incubated at 37°C for 14 days. The cells were then washed twice with

PBS and stained with 0.1% crystal violet for 15 minutes before counting. Clusters including at least 50 cells were counted as colonies. Three independent experiments were performed.

Antibody neutralization

To elucidate signal transduction pathways involving CXCR4 and Akt, Ca9-22 and CAL 27 cells with MRE11 overexpression were treated with CXCR4 ligand SDF-1 (500ng/mL) or CXCR4 neutralization antibody (5μg/mL) and incubated at 37°C for 48 hrs. The cell lysate was collected and protein level was analyzed by immunoblotting method.

Inhibition of MRE11 nuclease activity

The effects of MRE11 nuclease activity on cell survival and mobility were analyzed by MRE11 nuclease inhibitor mirin in Ca9-22 and CAL 27 cells with MRE11 overexpression at concentrations of 12.5 and 25μM for 24hrs. The cells were collected for further migration assay, and protein level analyzed by immunoblotting method. MRE11 H129N mutant, a nuclease-deficient form of MRE11 [3], was constructed and infected into oral cancer cells according to the subsection of “Virus infection for MRE11 knockdown/overexpression” in the Methods section.

Microarray data collection and gene expression analyses

Total RNA from control and MRE11-knockdowned cells was isolated with the RNeasy Mini kit. Synthesis of cRNA from total RNA and hybridization/scanning of microarrays were performed with Affymetrix GeneChip products (HGU133A) as described in the GeneChip manual. Normalization of the raw gene expression data, quality control checks, and subsequent analyses were performed with the open source R-project statistical (http://www.r-project.org/) and Bioconductor packages. After RMA normalization, the t statistic was used to generate a ranked list of differentially expressed genes. GSEA and IPA pathway analyses were performed with this preranked list.

RT^2 profiler PCR array

RNA from samples was extracted with the RNeasy Microkit (Qiagen). Reverse transcription and realtime PCR were performed with proprietary kits and reagents from Qiagen according to the manufacturer’s instructions. 84 key genes from human stem cell signaling PCR array (Qiagen; #PAHS-047Z) or Human Epithelial to Mesenchymal Transition (EMT) PCR array (Qiagen; #PAHS-090Z) were simultaneously assayed with the RT2 Profiler PCR array plate (Qiagen).

***In vivo* oral cancer cell invasiveness in zebrafish**

According to the 3Rs, we minimized the number of animals used in our experiments. Experiments involving zebrafish were approved by the Institutional Animal Care and

Use Committee of Kaohsiung Medical University (IACUC Approval No: 107023). Zebrafish were randomized into several groups. Embryos of zebrafish (strain *fli1:EGFP* from the Taiwan Zebrafish Core Facility; http://tzcf-hdmrc.org/) were generated by natural pairwise mating and the xenograft procedure was based on previous reports [4]. In brief, oral cancer cells with overexpressed or knockdowned MRE11 expression were labeled with fluorescent probe DiI (Life Technologies, Grand Island, NY, USA), and implanted into the perivitelline cavity of 2-day-old zebrafish embryos through microinjection. After confirmation of the localized DiI-labeled cell mass at the injection site, the zebrafish embryos were transferred to fresh water and maintained at 32.5°C for 48 h, and cell invasion was determined by visualizing dissemination of the DiI-labeled cells from the injection site under Nikon Eclipse Ti-S microscope (Tokyo, Japan).

*In vivo* orthotopic tumor growth and distant metastasis in mice

According to the 3Rs, we minimized the number of animals used in our experiments. Six-week-old male SCID(NOD.CB17-*Prkdc*^{scid}/JNarl) mice were obtained from the National Laboratory Animal Center of Taiwan (http://www.nlac.org.tw/), with approval of all experiments by the Institutional Animal Care and Use Committee of Kaohsiung Medical University (IACUC Approval No: 106094). Mice were randomized into several groups.

For orthotopic tumor model, oral cancer cells (5×10^5 cells) resuspended in 100 μL of normal saline were injected into mice through an intrabuccal route. After six weeks, all mice were sacrificed and orthotopic tumors collected for tumor weight measurement and immunohistochemical analysis.

For distant (lung) metastasis in SCID mice, oral cancer cells (2.5×10^5 cells) with luciferase expression were injected intravenously through tail vein, and distant (lung) metastasis was checked by an *In vivo* imaging system (IVIS) as described below. To explore the treatment strategies for MRE11-overexpressing oral cancers *in vivo*, oral cancer cells CAL 27 (2.5×10^5 cells) were injected intravenously through tail vein, followed by intravenous injection of CXCR4 neutralizing antibody or mouse IgG (1μg antibody per 1g body weight). CXCR4 neutralizing antibody or mouse IgG were injected twice a week for 6 weeks. All mice were euthanized six weeks later and lungs collected for H&E staining and metastasis analysis. The distant (lung) metastasis was analyzed by calculating H&E staining results.

***In vivo* imaging system (IVIS)**

Six-week old male immunodeficient mice were purchased from National Laboratory Animal Center (NLAC, Taiwan) and injected with $2.5x10^5$ MRE11OE/Luc cells suspended in 100 μL PBS via tail vein. Cancer metastasis was monitored using IVIS-50 optical imaging system (PerkinElmer) after 150 mg/kg luciferin injection

periodically. Before sacrifice, the mice were injected with luciferin, and mice organs were collected for IVIS and histology analyses.

Statistical analysis

The estimated odds ratio (OR) was used to summarize the relationship between MRE11 expression and clinicopathological features of patients. The Cox proportional hazard model was used to evaluate the association between clinicopathologic factors and survival for multivariate analysis. In the multivariate analysis, variables showing *p* values of less than 0.05 in the univariate analysis were included as covariates. The OS and DFS were calculated using the Kaplan-Meier method, and survival curves were compared by log-rank test. Two-sided Student t test was used for in vitro and in vivo studies. All statistical analyses were performed using JMP version 10.0.1 for Windows (SAS Institute, Cary, NC, USA) and p values less than 0.05 were considered statistically significant.

References

1 Chen LC, Chung IC, Hsueh C, Tsang NM, Chi LM, Liang Y *et al*. The antiapoptotic protein, FLIP, is regulated by heterogeneous nuclear ribonucleoprotein K and correlates with poor overall survival of nasopharyngeal carcinoma patients. Cell death and differentiation 2010; 17: 1463-1473.

2 Chuang FH, Hsue SS, Wu CW, Chen YK. Immunohistochemical expression of RANKL, RANK, and OPG in human oral squamous cell carcinoma. Journal of oral pathology & medicine : official publication of the International Association

of Oral Pathologists and the American Academy of Oral Pathology 2009; 38: 753-758.

3 Hoa NN, Shimizu T, Zhou ZW, Wang ZQ, Deshpande RA, Paull TT *et al*. Mre11 Is Essential for the Removal of Lethal Topoisomerase 2 Covalent Cleavage Complexes. Molecular cell 2016; 64: 580-592.

4 Lee SL, Rouhi P, Dahl Jensen L, Zhang D, Ji H, Hauptmann G *et al*. Hypoxia-induced pathological angiogenesis mediates tumor cell dissemination, invasion, and metastasis in a zebrafish tumor model. Proceedings of the National Academy of Sciences of the United States of America 2009; 106: 19485-19490.

5 Lee YC, Yang YH, Su JH, Chang HL, Hou MF, Yuan SS. High visfatin expression in breast cancer tissue is associated with poor survival. Cancer epidemiology, biomarkers & prevention : a publication of the American Association for Cancer Research, cosponsored by the American Society of Preventive Oncology 2011; 20: 1892-1901.

6 Rhodes DR, Yu J, Shanker K, Deshpande N, Varambally R, Ghosh D *et al*. ONCOMINE: a cancer microarray database and integrated data-mining platform. Neoplasia (New York, NY) 2004; 6: 1-6.

7 Sarbia M, Stahl M, Fink U, Willers R, Seeber S, Gabbert HE. Expression of apoptosis-regulating proteins and outcome of esophageal cancer patients treated by combined therapy modalities. Clinical cancer research : an official journal of the American Association for Cancer Research 1998; 4: 2991-2997.

8 Verschuur HP, Irish JC, O'Sullivan B, Goh C, Gullane PJ, Pintilie M. A matched control study of treatment outcome in young patients with squamous cell carcinoma of the head and neck. The Laryngoscope 1999; 109: 249-258.

9 Wang SH, Chang JS, Hsiao JR, Yen YC, Jiang SS, Liu SH *et al*. Tumour cell-derived WNT5B modulates in vitro lymphangiogenesis via induction of partial endothelial-mesenchymal transition of lymphatic endothelial cells. Oncogene 2017; 36: 1503-1515.

10 Yuan SS, Hou MF, Hsieh YC, Huang CY, Lee YC, Chen YJ *et al*. Role of MRE11 in cell proliferation, tumor invasion, and DNA repair in breast cancer.

J Natl Cancer Inst 2012; 104: 1485-1502.

Supplementary data

Figure S1. MRE11 expression and oral cancer cell behavior. (A) Transwell migration ability in various oral cancer cell lines at 24 hrs and 48 hrs. **(B)** MRE11 protein expression in OEC-M1 oral cancer cells after infection with different lentiviral knockdown constructs, as determined by immunoblotting. **(C)** MRE11 mRNA expression in HSC-3 oral cancer cells after infection with clone 1 lentiviral knockdown construct, as determined by qRT-PCR. **(D)** MRE11 protein expression in oral cancer cells after lentiviral knockdown and overexpression, as determined by immunoblotting. **(E)** Decreased oral cancer cell viability after MRE11 knockdown, as determined by XTT cell viability assay. **(F)** The expression of p-AKT and p-ERK was decreased after MRE11 knockdown in oral cancer cells. **(G)** Decreased oral cancer cell colony formation after MRE11 knockdown. **(H)** The dosage effect of mirin on CAL 27 oral cancer cell death after treatment for 72 hours

Figure S2. The effect of MRE11 knockdown and overexpression on colony formation in oral cancer cells 7 days after ionizing radiation exposure.

Figure S3. The effect of MRE11 expression on chemoresistance. (A) The effect of MRE11 knockdown on oral cancer cell viability, as determined by XTT cell viability

assay after CDDP treatment. **(B)** The effect of MRE11 knockdown on comet tail formation, an indicator for DSBs, in oral cancer cells after CDDP treatment. **(C)** The effect of MRE11 knockdown on γH2AX expression, an indicator for DSBs, in oral cancer cells after CDDP treatment. **(D)** The effect of MRE11 knockdown on oral cancer cell apoptosis, as determined by TUNEL positivity, after CDDP treatment. **(E)** The effect of MRE11 knockdown on oral cancer cell apoptosis, as determined by Annexin V positivity, after CDDP treatment. **(F)** The effect of MRE11 expression, determined by IHC, on overall survival of oral cancer patients after chemotherapy.

Figure S4. CXCR4 mRNA expression in MRE11-overexpressing oral cancer cells. **(A)** The evaluation of tumor metastasis-associated gene expression profiles in MRE11-overexpressing oral cancer cells was carried out using RT^2 Profiler PCR Array– Human Tumor Metastasis (SABioscience). **(B)** CXCR4 mRNA expression in MRE11-overexpressing cells without or with CXCR4 silencing.

Figure S5. MRE11 regulates the expression of FOXA2 and E-cadherin. **(A)** In MRE11-knockdowned oral cancer cells, FOXA2 moved to the nucleus. **(B)** A negative correlation between MRE11 and E-cadherin expression in oral cancer tissues, as determined by IHC. **(C)** A positive correlation between FOXA2 and E-cadherin

expression in oral cancer tissues. **(D)** Correlation between MRE11, CXCR4 and FOXA2 expression using microarray datasets for oral cancer retrieved from ONCOMINE Cancer Profiling Database (https://www.oncomine.org).

Figure S6. The effect of MRE11 knockdown on LN1-1 oral cancer cell viability and tumor growth. (A) The effect of MRE11 knockdown on the viability of LN1-1 cells. **(B)** The effect of MRE11 knockdown on transwell migration of LN1-1 cells. **(C)** The effect of MRE11 knockdown on total flux in LN1-1 orthotopic mouse model. **(D)** The effect of MRE11 knockdown on tumor volume in LN1-1 orthotopic mouse model.

Figure S7. Decreased expression of mesenchymal marker vimentin (A) and increased expression of epithelial marker E-cadherin (B) in the MRE11-knockdowned orthotopic mouse tumor model.

Figure S8. The effect of MRE11 expression in oral cancer cells on the expression of *p*-STAT3.

Figure S9. Correlation of MRE11 expression with expression of replication stress markers (A) γH2AX, (B) *p*-ATM and (C) *p*-ATR in oral cancer tissues, as

determined by immunohistochemistry analysis. The relationship between MRE11 expression and γH2AX, *p*-ATM and *p*-ATR expression in oral cancer tissues was examined using linear regression analysis.
