# Supplementary figures and images for: MRE11 promotes oral cancer progression through RUNX2/CXCR4/AKT/FOXA2 signaling in a nuclease-independent manner
Source: Oncogene. 2021 Apr 29;40(20):3510–32. doi: 10.1038/s41388-021-01698-5 (PMC8134045; doi:10.1038/s41388-021-01698-5)

## Slide 1
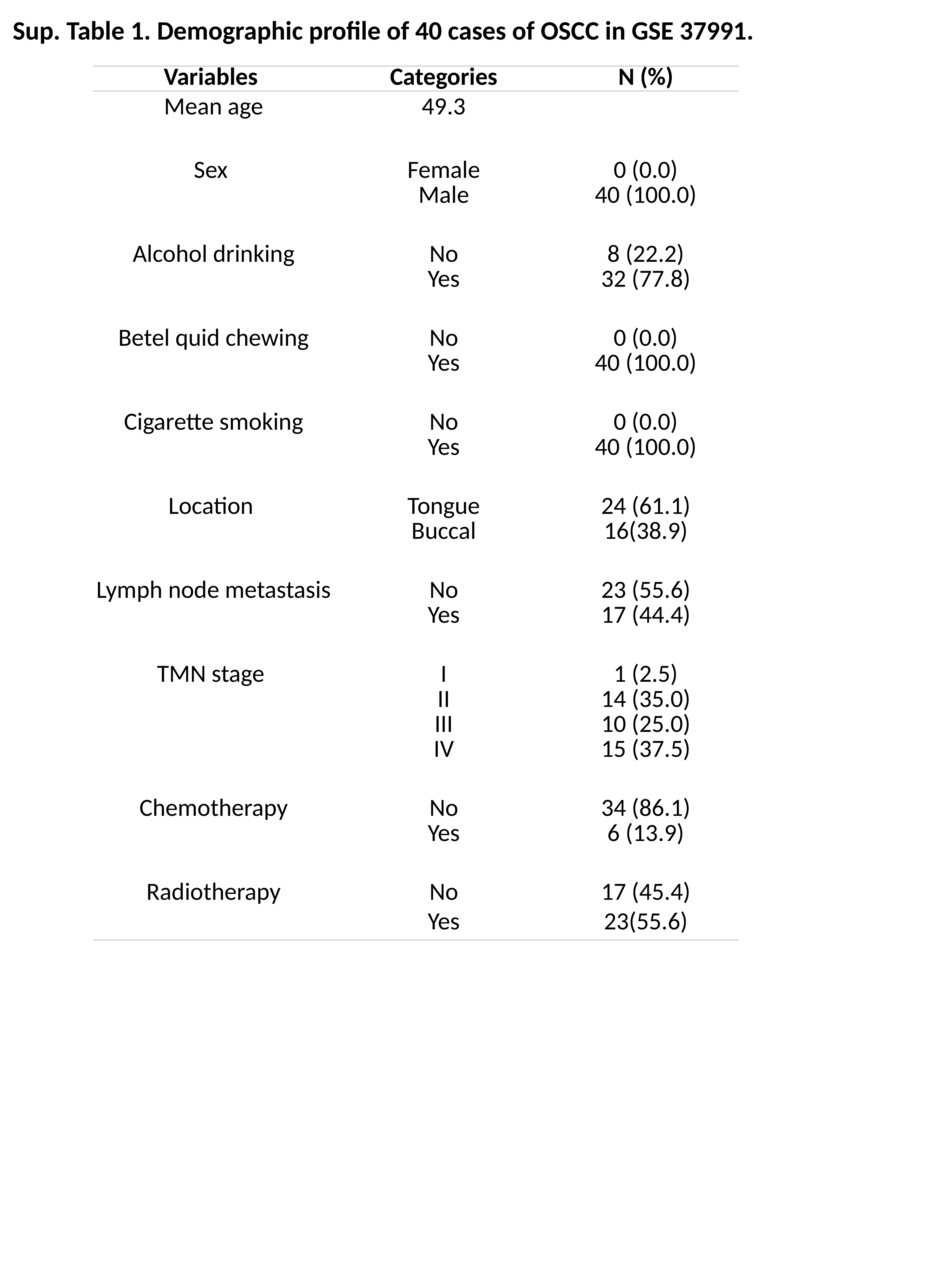

## Slide 2
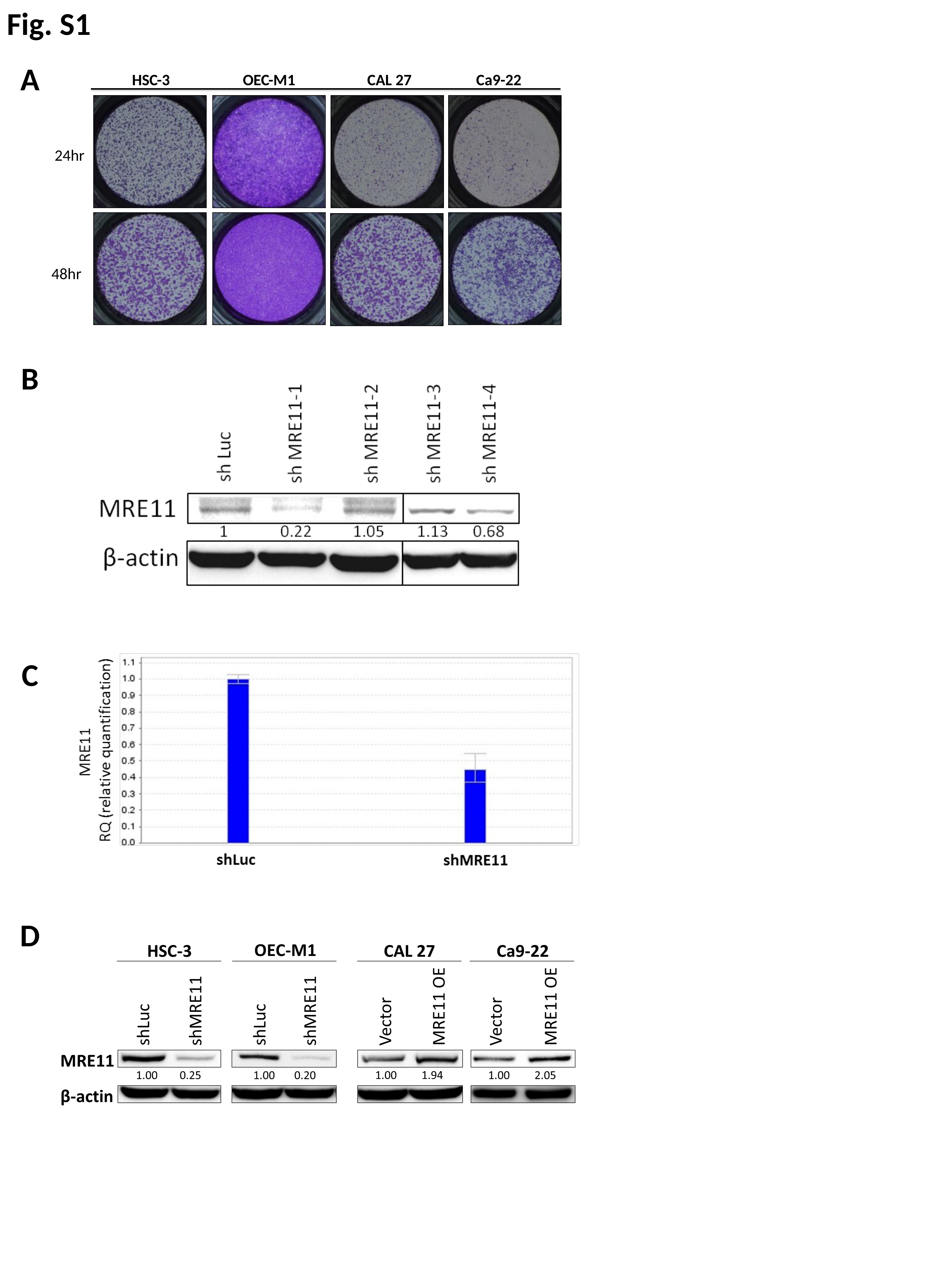

## Slide 3
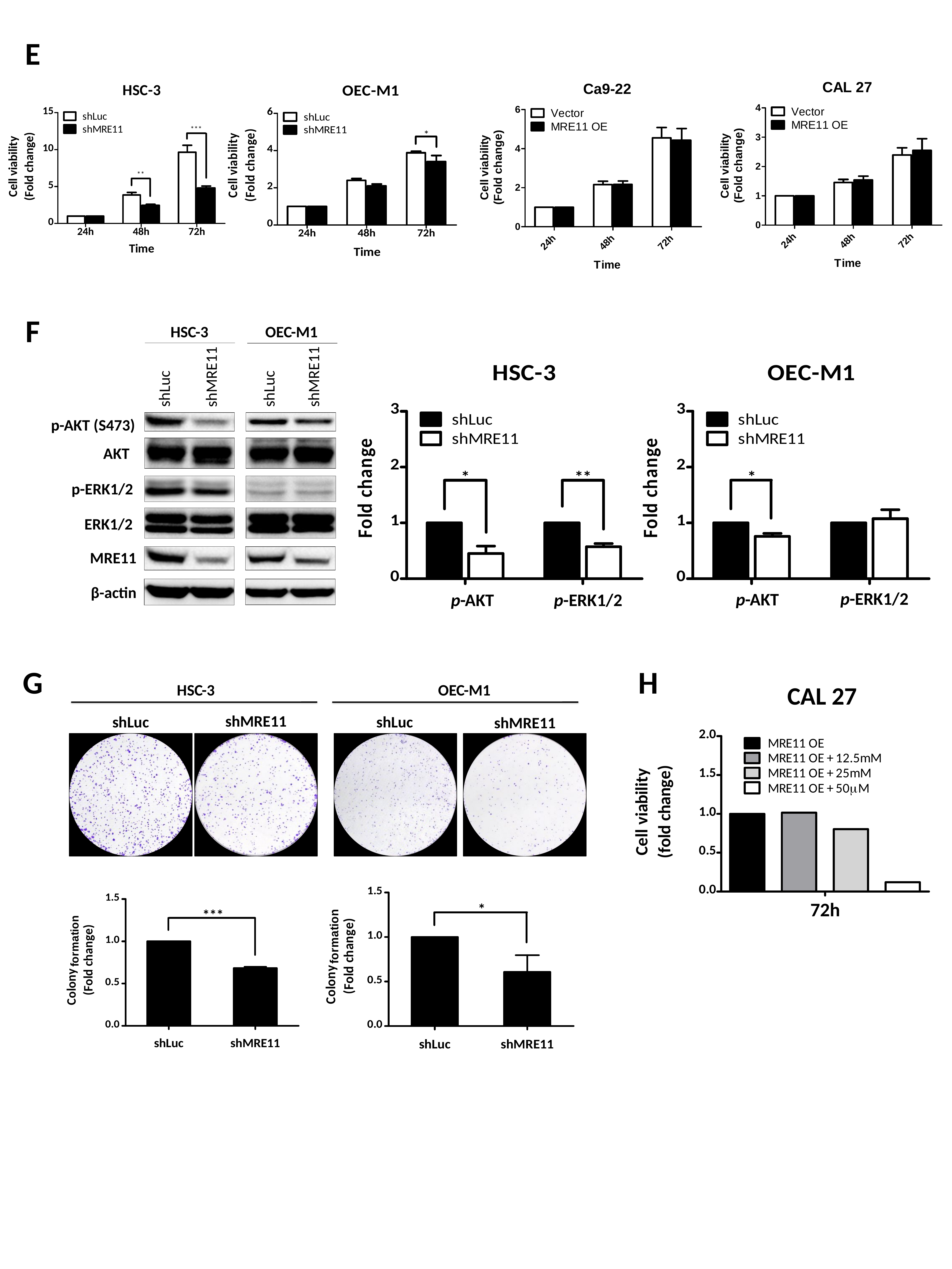

## Slide 4
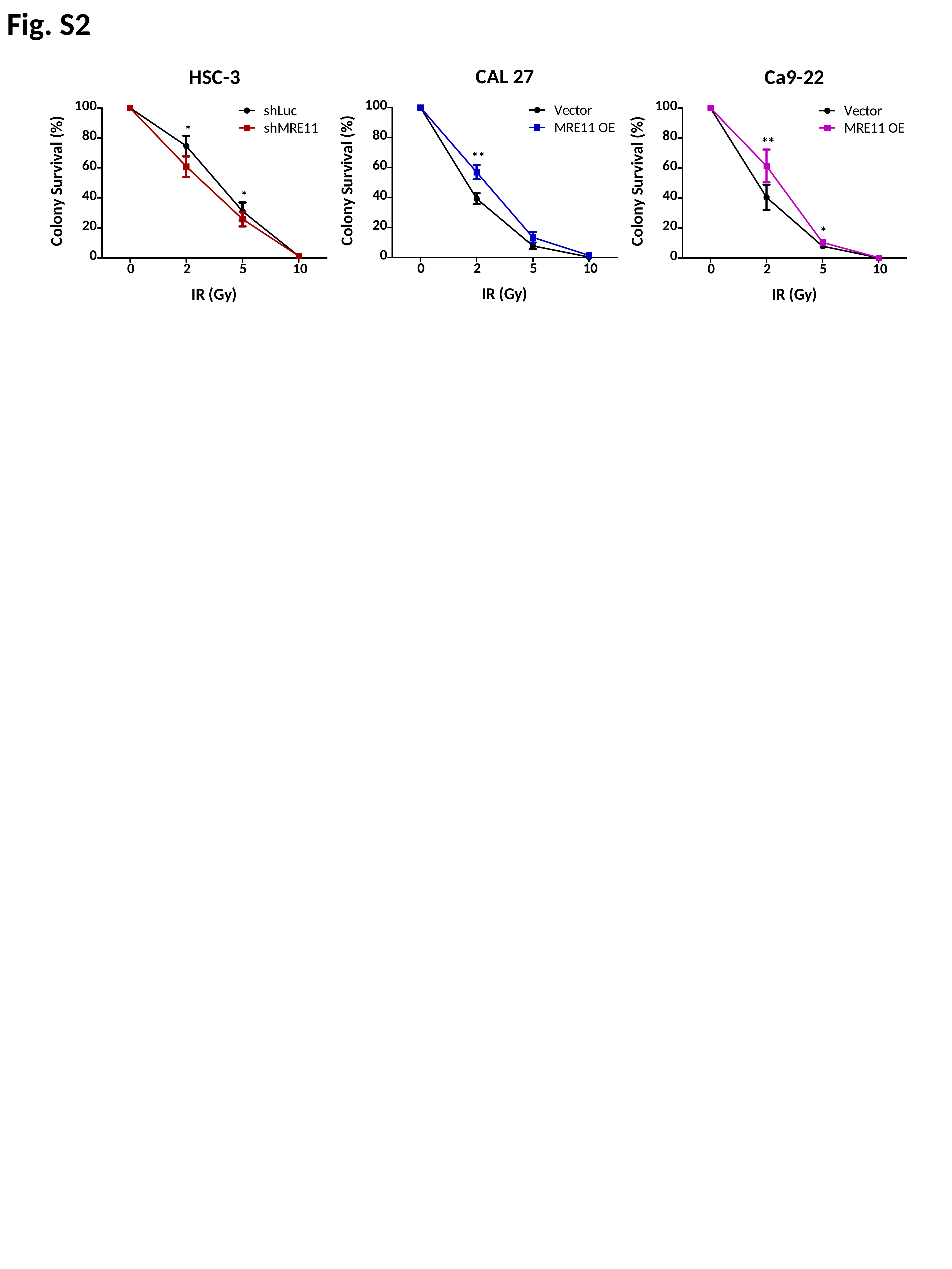

## Slide 5
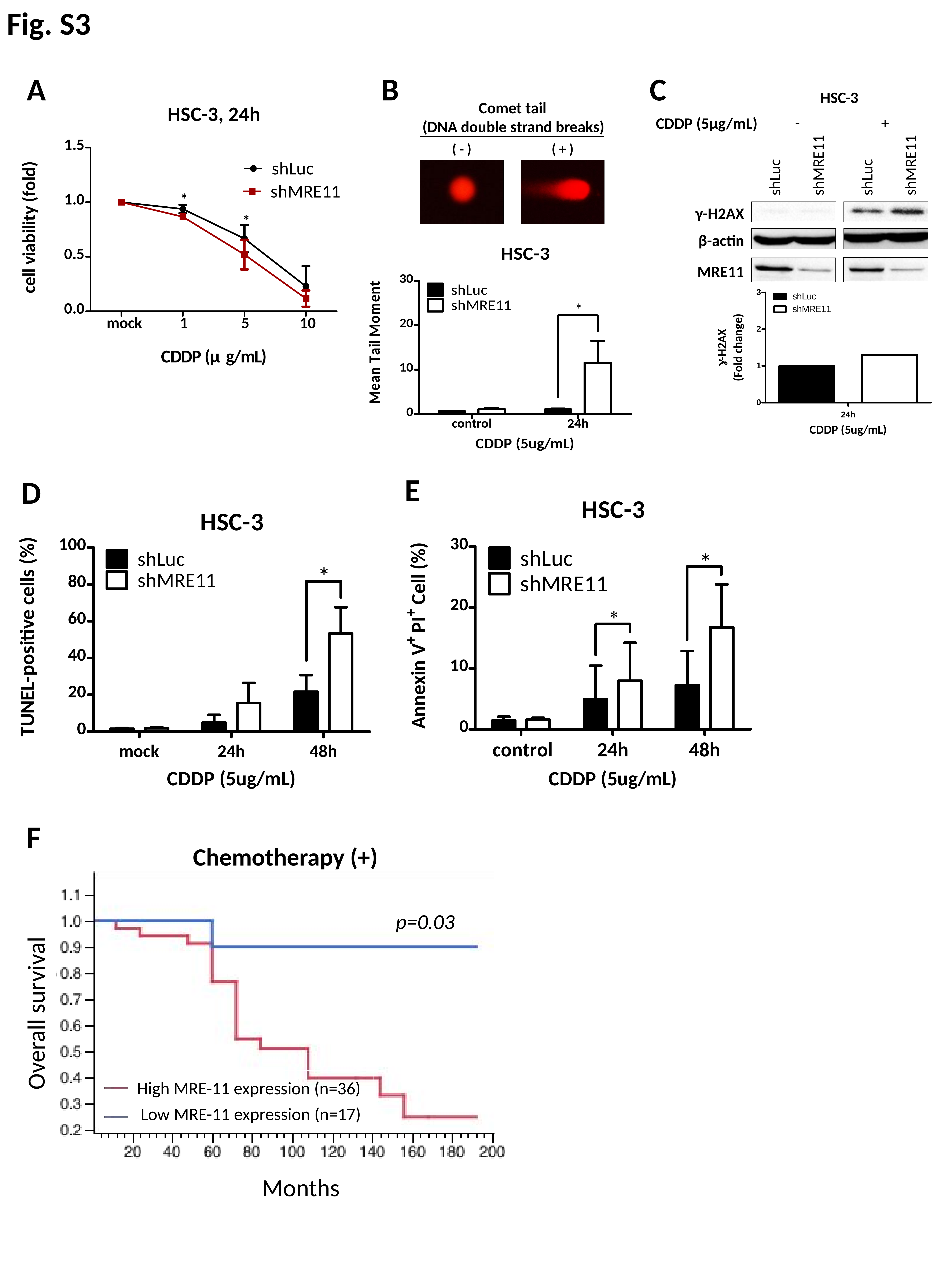

## Slide 6
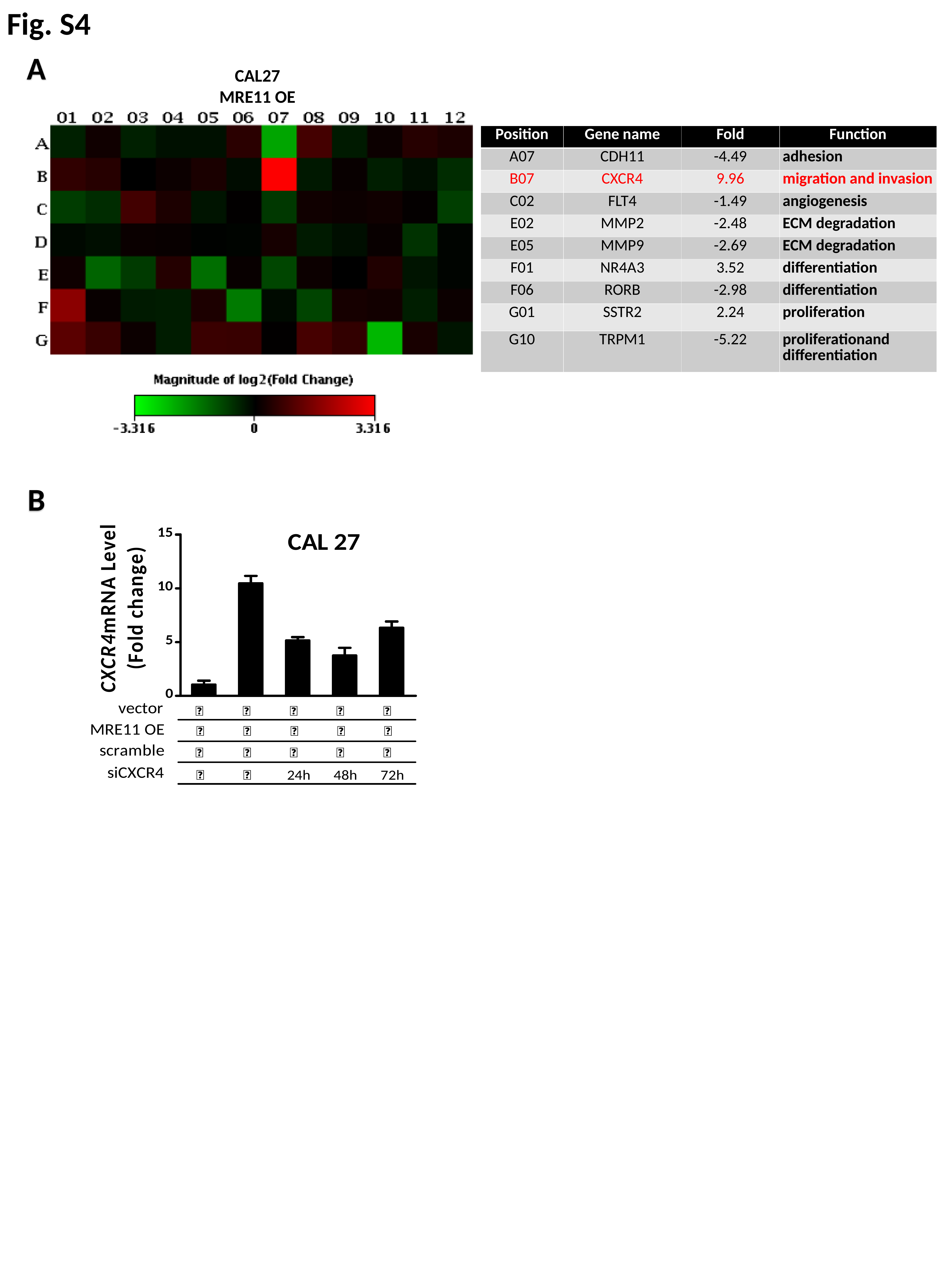

## Slide 7
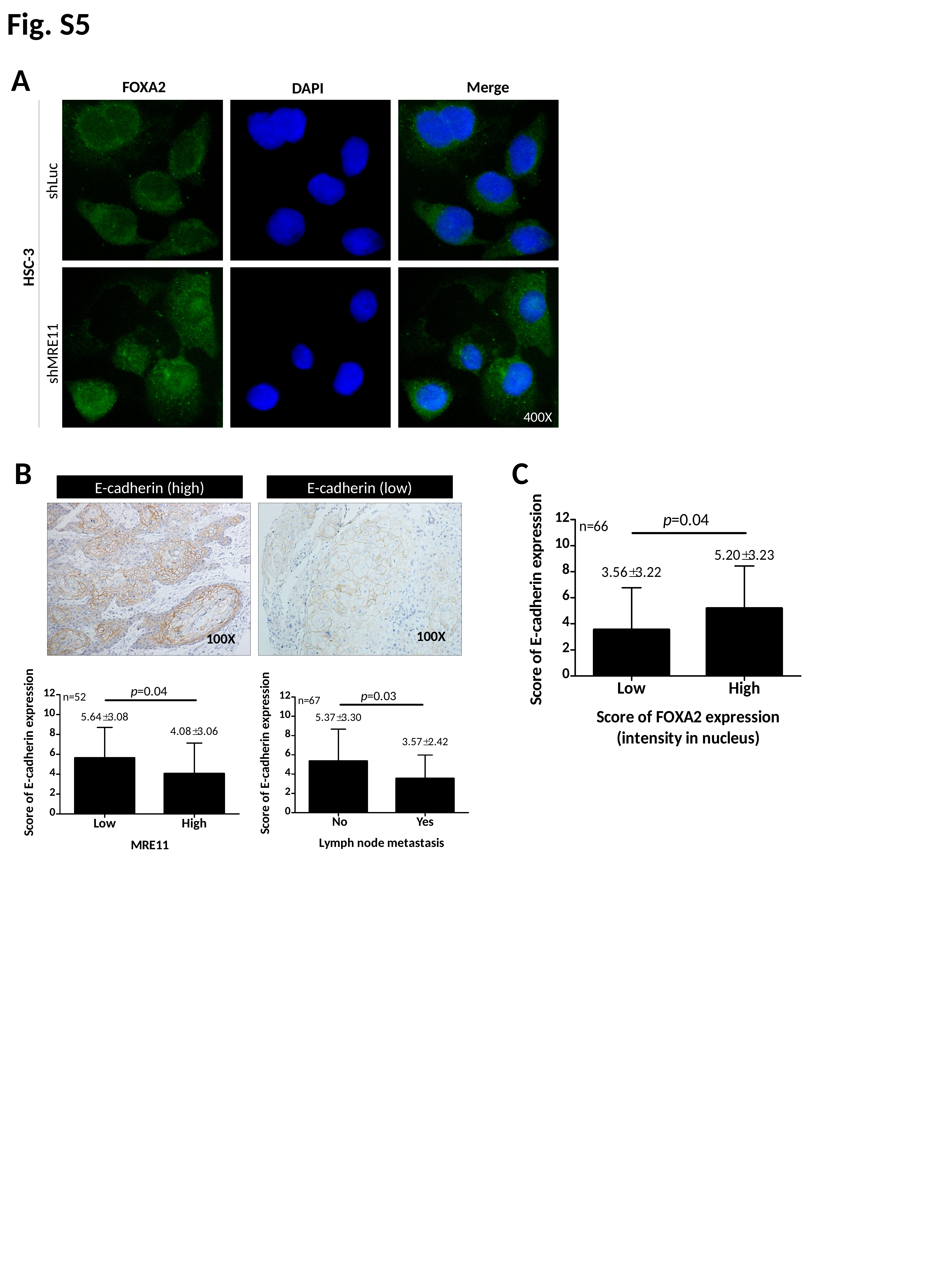

## Slide 8
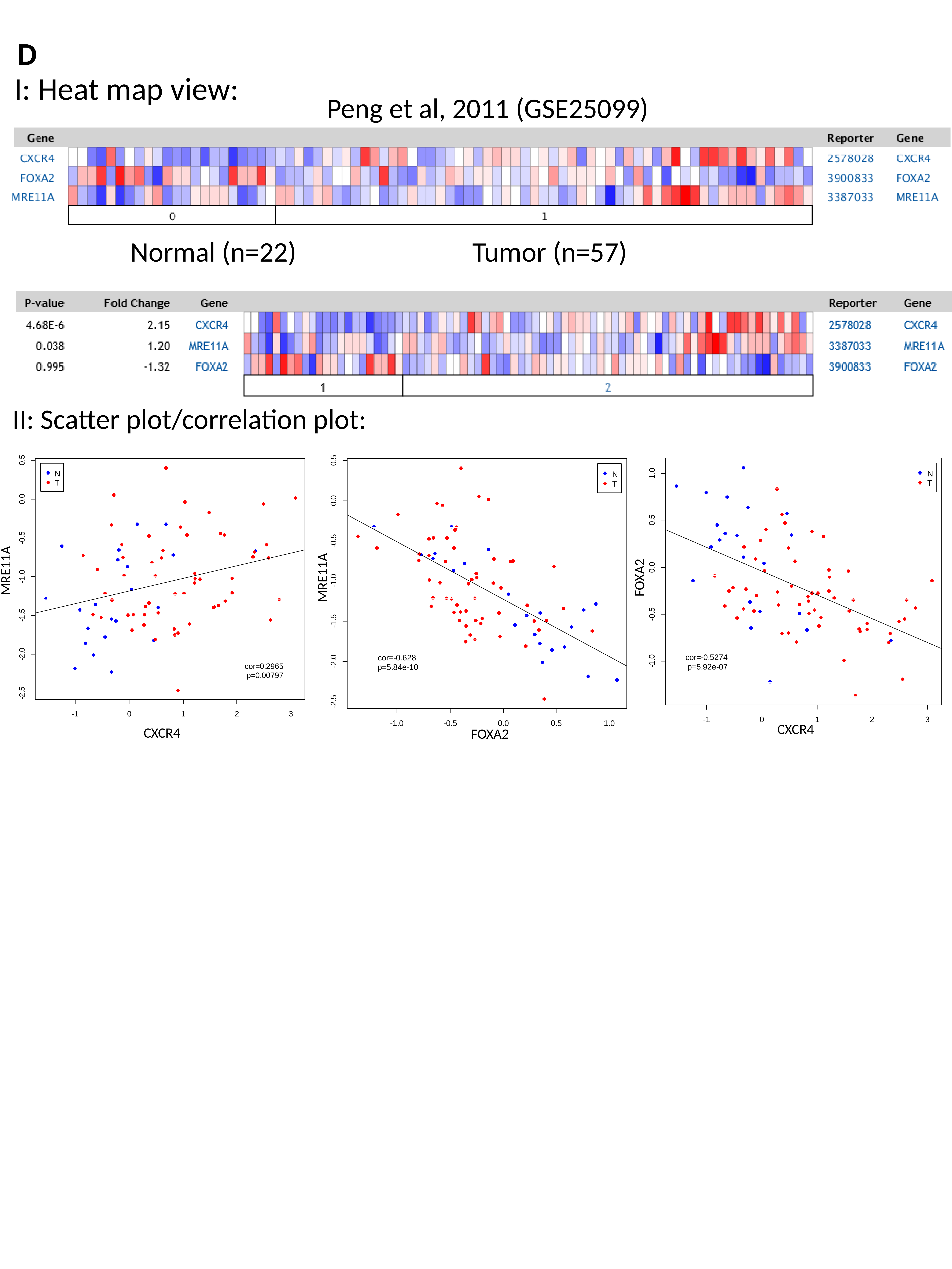

## Slide 9
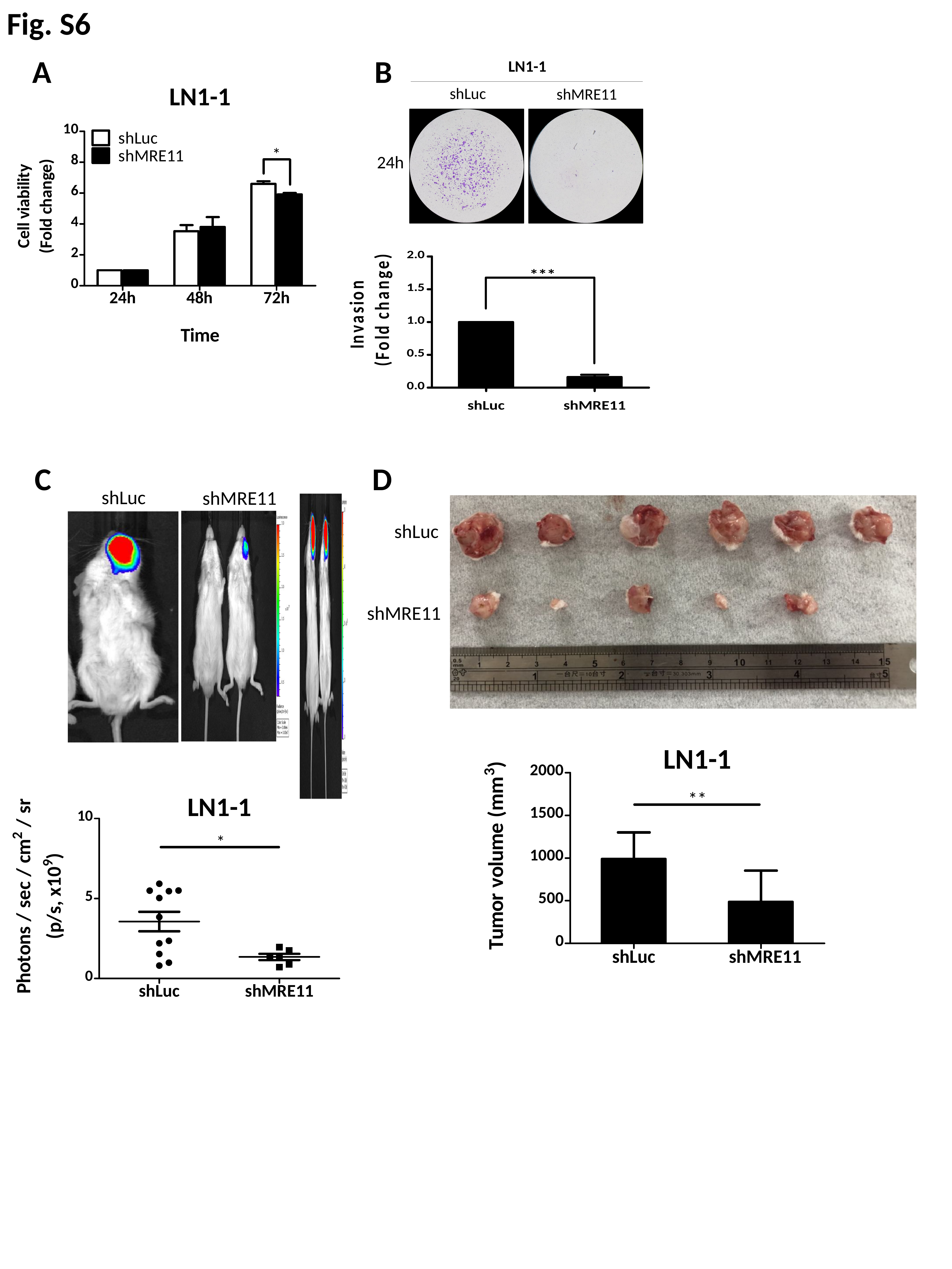

## Slide 10
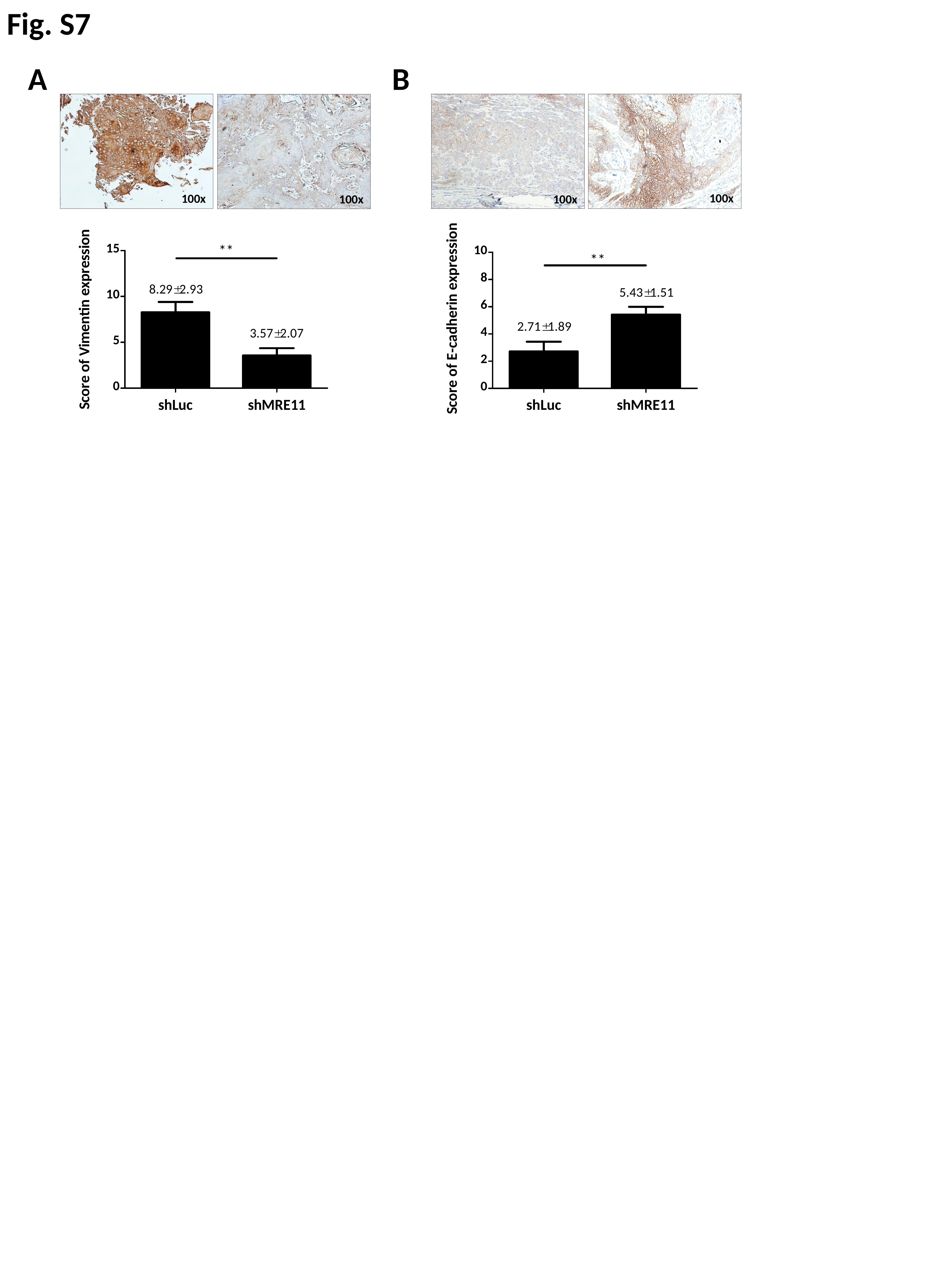

## Slide 11
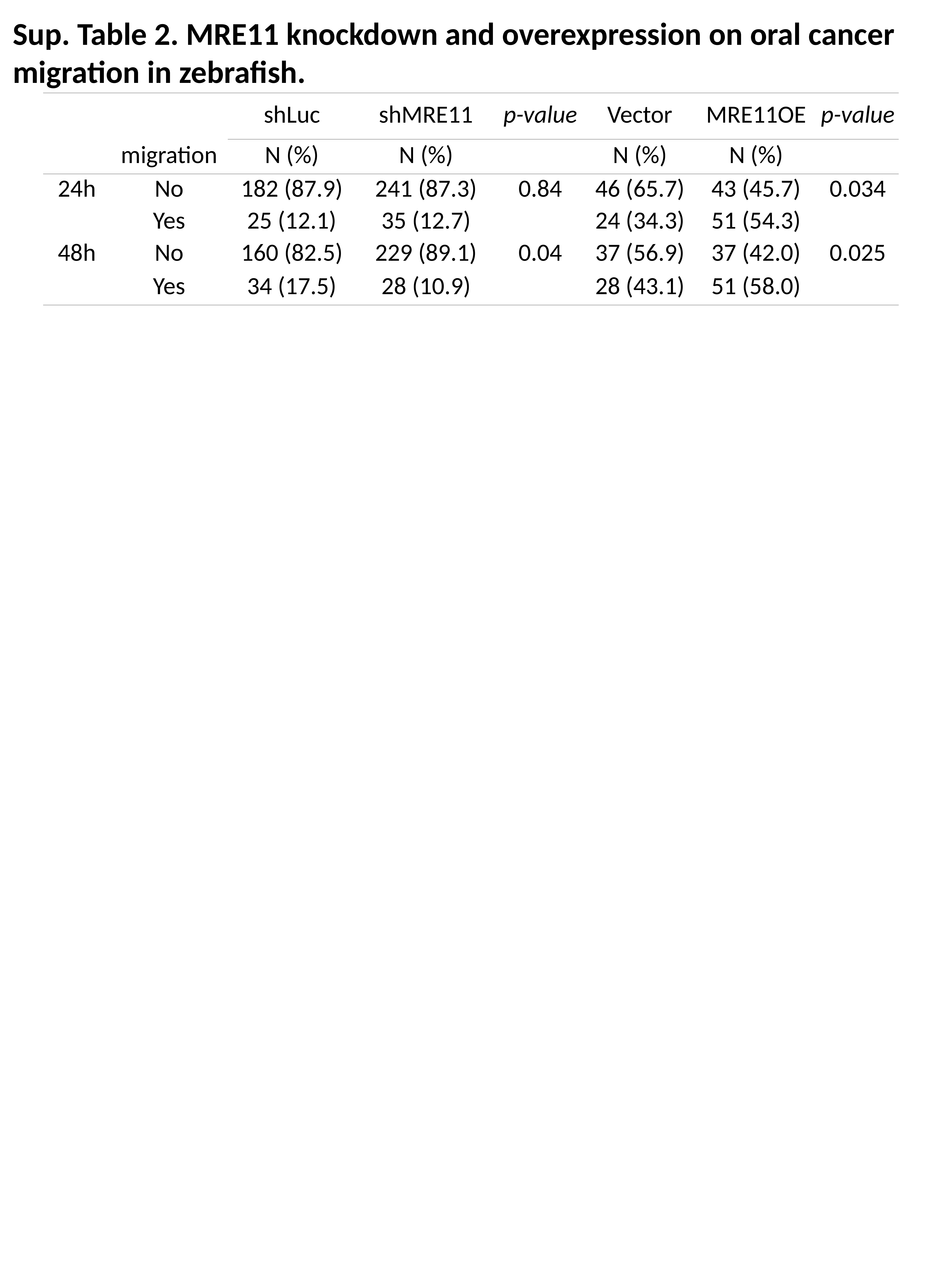

## Slide 12
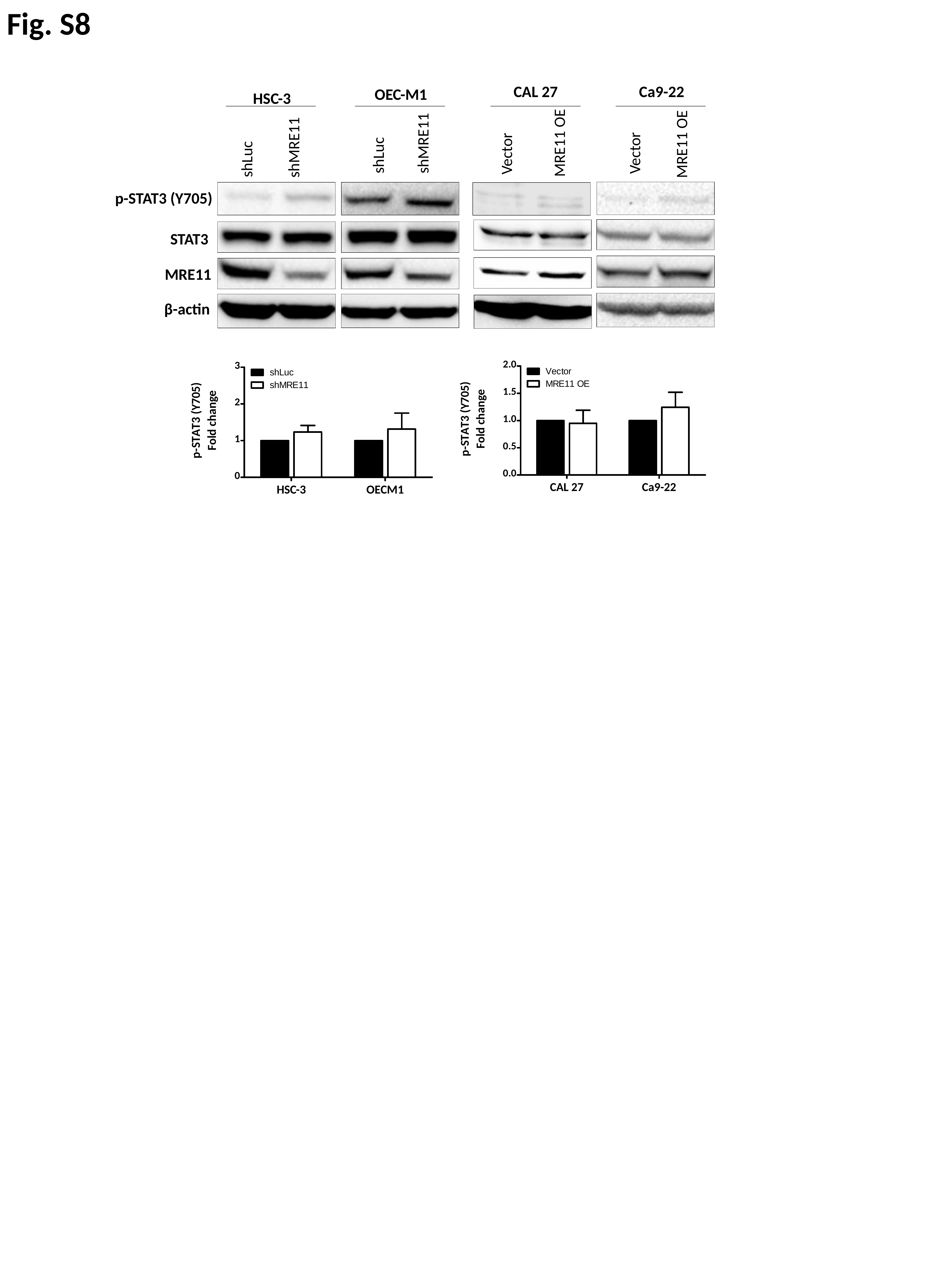

## Slide 13
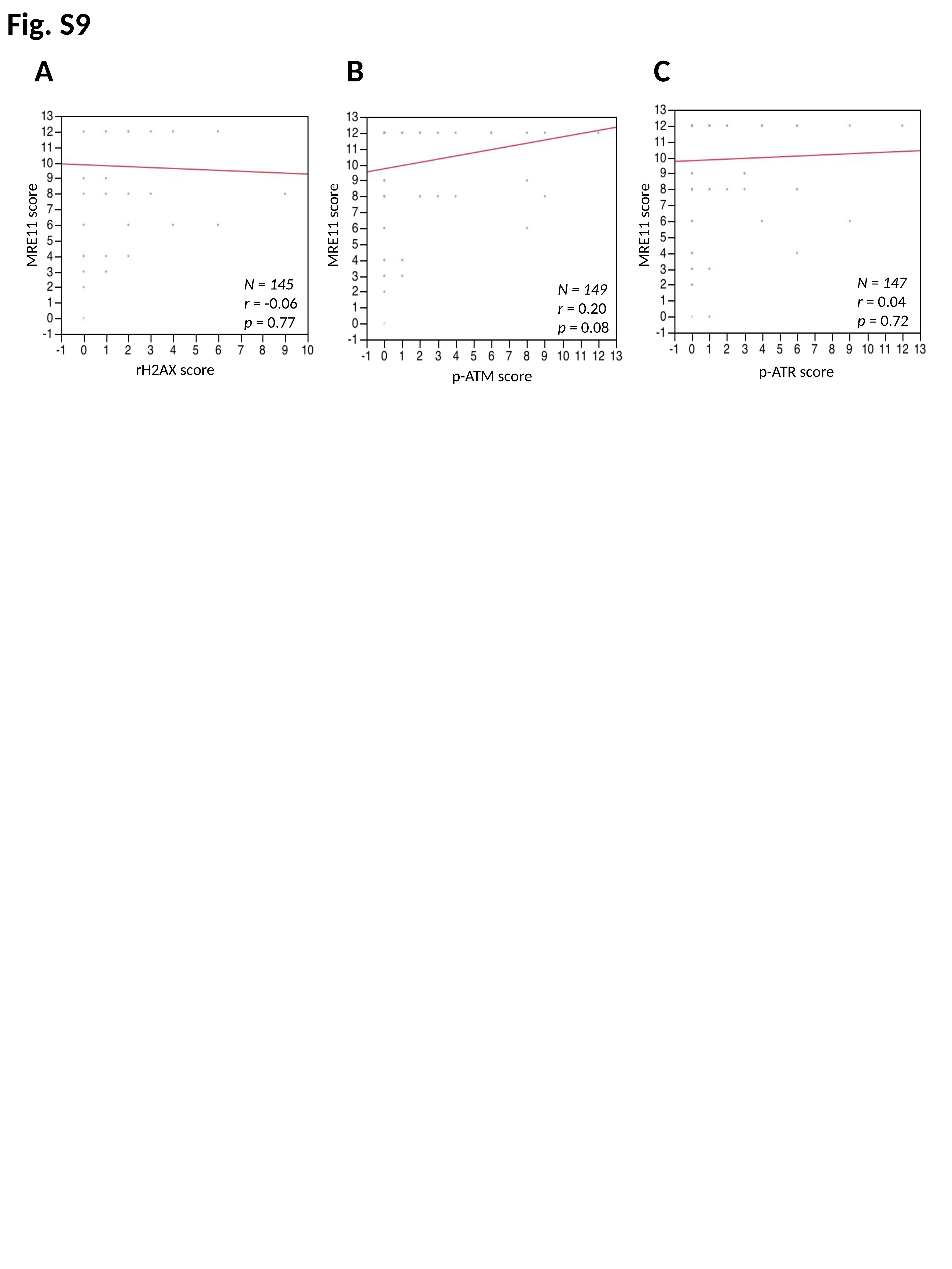

Supplement: Supplementary file 2 — Supplementary data [file 41388_2021_1698_MOESM2_ESM.pptx]
